# Labeling Strategies Matter for Super-Resolution Microscopy: A Comparison between HaloTags and SNAP-tags

**DOI:** 10.1016/j.chembiol.2019.01.003

**Published:** 2019-04-18

**Authors:** Roman S. Erdmann, Stephanie Wood Baguley, Jennifer H. Richens, Rebecca F. Wissner, Zhiqun Xi, Edward S. Allgeyer, Sheng Zhong, Alexander D. Thompson, Nicholas Lowe, Richard Butler, Joerg Bewersdorf, James E. Rothman, Daniel St Johnston, Alanna Schepartz, Derek Toomre

**Affiliations:** 1 Department of Cell Biology, Yale University School of Medicine, 333 Cedar Street, New Haven, CT, USA; 2 Department of Chemistry, Yale University, 225 Prospect Street, New Haven, CT, USA; 3 Wellcome Trust/Cancer Research UK Gurdon Institute, University of Cambridge, Tennis Court Road, Cambridge CB2 1QN, UK; 4 Department of Biomedical Engineering, Yale University, 55 Prospect Street, New Haven, CT, USA

**Keywords:** self-labeling proteins, live-cell imaging, super-resolution microscopy, HaloTag, SNAP-tag, STED, fluorophores, nanoscopy, microscopy

## Abstract

Super-resolution microscopy requires that subcellular structures are labeled with bright and photostable fluorophores, especially for live-cell imaging. Organic fluorophores may help here as they can yield more photons—by orders of magnitude—than fluorescent proteins. To achieve molecular specificity with organic fluorophores in live cells, self-labeling proteins are often used, with HaloTags and SNAP-tags being the most common. However, how these two different tagging systems compare with each other is unclear, especially for stimulated emission depletion (STED) microscopy, which is limited to a small repertoire of fluorophores in living cells. Herein, we compare the two labeling approaches in confocal and STED imaging using various proteins and two model systems. Strikingly, we find that the fluorescent signal can be up to 9-fold higher with HaloTags than with SNAP-tags when using far-red rhodamine derivatives. This result demonstrates that the labeling strategy matters and can greatly influence the duration of super-resolution imaging.

## Highlights

• Systematic comparison of SNAP versus Halo tag labeling by confocal and STED microscopy
• Target proteins, fluorophores, and model systems are compared
• Large differences in Halo versus SNAP intensity with silicon rhodamine fluorophores
• Guidelines for one- and two-color super-resolution imaging are provided

## Teaser

Self-labeling proteins leverage the superior photophysical properties of organic fluorophores and are the method of choice for live-cell nanoscopy. Comparing SNAP-tags and HaloTags, Erdmann et al. show that Halo tagging with silicon rhodamine fluorophores provides brighter labeling for confocal and STED nanoscopy.

## Introduction

Super-resolution fluorescence microscopy, also called “nanoscopy,” enables the visualization of cellular structures beyond the diffraction limit of light (Fornasiero and Opazo, 2015, Hell, 2007, Huang et al., 2009, Toomre and Bewersdorf, 2010, van de Linde et al., 2012). However, unlike electron microscopy, whose application is limited to fixed cells, nanoscopy enables live-cell imaging to study cellular dynamics in unprecedented spatial detail. Green fluorescent protein (GFP) and its spectral variants (Uno et al., 2015) have revolutionized biology, as they allow genetically encoded labeling, but they possess mediocre photophysical properties, generally emitting fewer photons than the best organic dyes by one or two orders of magnitude (Dempsey et al., 2011, Fernandez-Suarez and Ting, 2008). While this deficiency may not be limiting for a single confocal image or even an image stack, the demands of nanoscopy are much greater, as every photon counts to obtain the highest resolution. Similarly, for 3D time-lapse fluorescence microscopy (4D imaging), which involves the acquisition of large datasets, correspondingly brighter and more stable fluorophores are required to study the volumetric dynamics of cells and tissues over longer timescales.

For both super-resolution imaging and 4D imaging, organic fluorophores are highly appealing because of their brightness and photostability (Dempsey et al., 2011, Fernandez-Suarez and Ting, 2008). Organic fluorophores can be attached to proteins by combining click chemistry with unnatural amino acid incorporation (Lang et al., 2012a, Lang et al., 2012b). A second option is the direct coupling to proteins in live cells by using self-labeling proteins such as SNAP-tags (Keppler et al., 2003) (or a variant called CLIP-tag; Gautier et al., 2008) and HaloTags (Los et al., 2008). Alternatively, labeling can be achieved by combining click chemistry and self-labeling proteins (Murrey et al., 2015). Like GFP, these self-labeling SNAP-tags and HaloTags can be expressed as fusion proteins (Hinner and Johnsson, 2010) and selectively reacted with the substrates benzylguanine (BG) and chloralkane (CA), respectively, which are tagged with organic fluorophores. While this labeling strategy is becoming increasingly popular for super-resolution imaging (Bottanelli et al., 2016, Bottanelli et al., 2017, Grimm et al., 2015, Stagge et al., 2013, Xue et al., 2015), especially since several commercial fluorescent SNAP and HALO ligands are available, it is unclear if these different tags influence the fluorescence properties of organic dyes, thereby possibly affecting image quality.

Herein, by conducting quantitative comparisons of SNAP and Halo tagging, we present strong evidence that the tag, its molecular targeting location, and its environment can have a major impact on the brightness of the introduced fluorophores. The difference in brightness can be striking—by nearly an order of magnitude—indicating that the labeling strategy matters greatly and can have a profound impact on image quality and duration by 4D confocal microscopy and stimulated emission depletion (STED) nanoscopy.

## Results and Discussion

### SiR Labeling of ST-Halo Tag Is Brighter Than that of ST-SNAP

**Figure 1:**
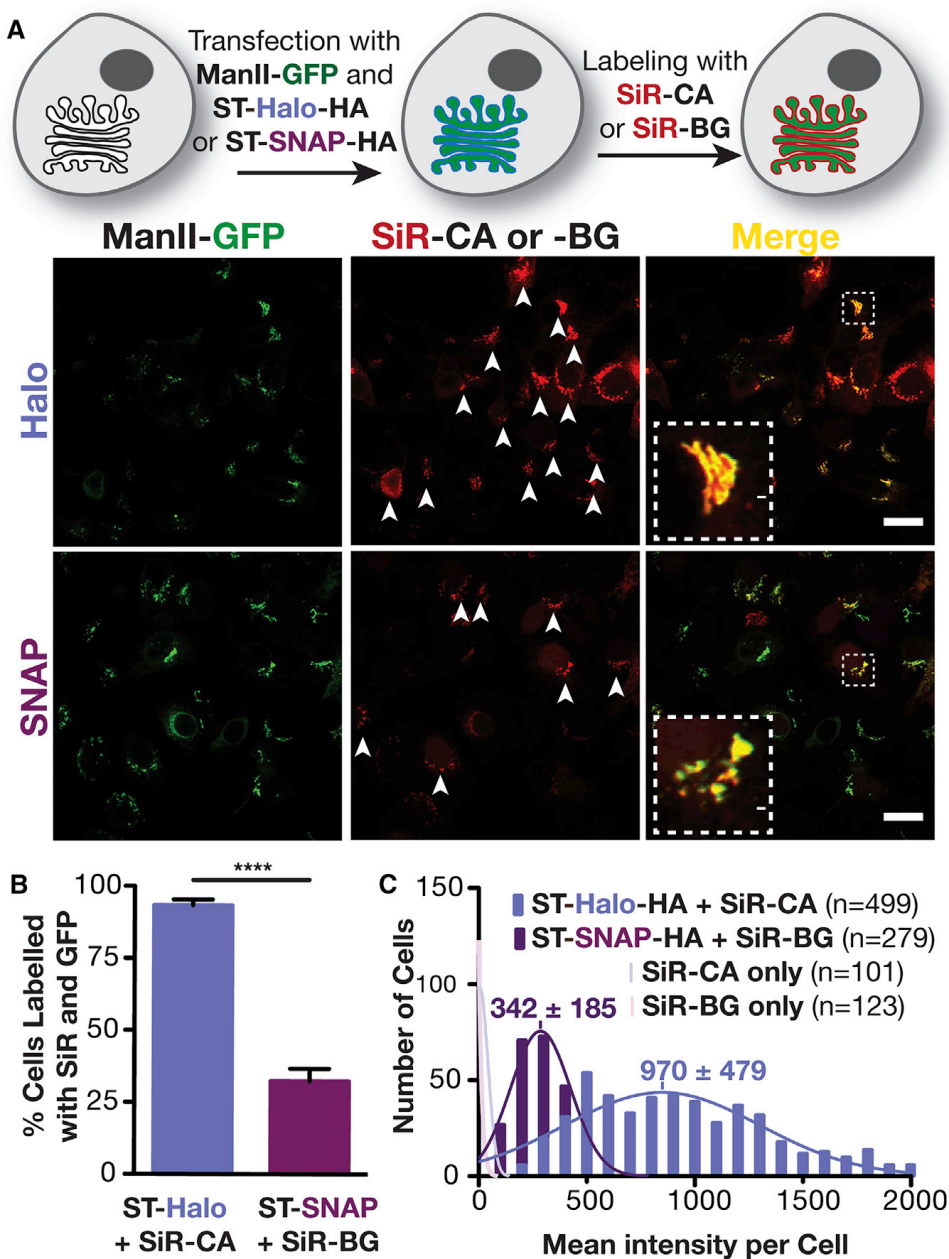
Comparison of Golgi Labeling with HaloTag and SNAP-tag Fusion Proteins of Sialyltransferase (A) Top: Scheme of the labeling procedure. Bottom: Confocal images of live HeLa cells that have been treated as described in the scheme above. The white arrowheads indicate cells that express ManII-GFP and have been labeled with SiR-CA or SiR-BG. Scale bar: 20 μm. (B) Quantification of cells expressing ManII-GFP that are positive for SiR from three independent experiments (ST-Halo, 740 cells in total; ST-SNAP, 837 cells in total). (C) Fluorescence intensity distribution of HeLa cells that were incubated with SiR-CA or SiR-BG and that are transiently expressing ST-Halo-HA, ST-SNAP-HA, or no fusion protein. The number of cells (n) analyzed is indicated in the plot.

We first compared HaloTag and SNAP-tag systems in cells transiently co-expressing mannosidase II (ManII)-GFP (Velasco et al., 1993) and sialyltransferase (ST; Kweon et al., 2004) fused to either the HaloTag or the SNAP-tag at its C terminus (Figure S1). Cells were labeled with Halo or SNAP ligands conjugated to the near far-red fluorophore silicon rhodamine (SiR): SiR-CA and SiR-BG for HaloTags and SNAP-tags, respectively (Lukinavicius et al., 2013) (Figure 1A). As expected, both Halo- and SNAP-tagged ST colocalized with ManII-GFP at the Golgi apparatus, as visualized by confocal microscopy. However, the fluorescence of SiR was strikingly much brighter for the Halo-tagged protein than for the SNAP-tagged one. A visual inspection showed that 93% of ManII-GFP-expressing cells were co-labeled with SiR for the HaloTag condition, whereas only 32% of GFP-tagged cells showed co-labeling for SNAP-tag (Figure 1B), suggesting that the majority of SNAP-tag cells were unlabeled with SiR. However, a quantitative analysis of hundreds of cells indicated that most SNAP-tag cells were indeed labeled, because they were clearly brighter than control cells lacking SNAP-tags and HaloTags, which could not be labeled with SiR (faint pink distributions in Figure 1C). The SNAP-tag cells were just much more dimly labeled than the HaloTag cells. The mean intensity of SiR with HaloTag was 2.8-fold brighter than with SNAP-tag, with both labeling systems showing expected Gaussian distributions of SiR intensities (Figure 1C). This surprising difference in brightness between the two popular tagging systems was intriguing and warranted further investigation.

### Ruling Out Transfection Efficiency, Substrate Permeability, and Expression Levels

**Figure 2:**
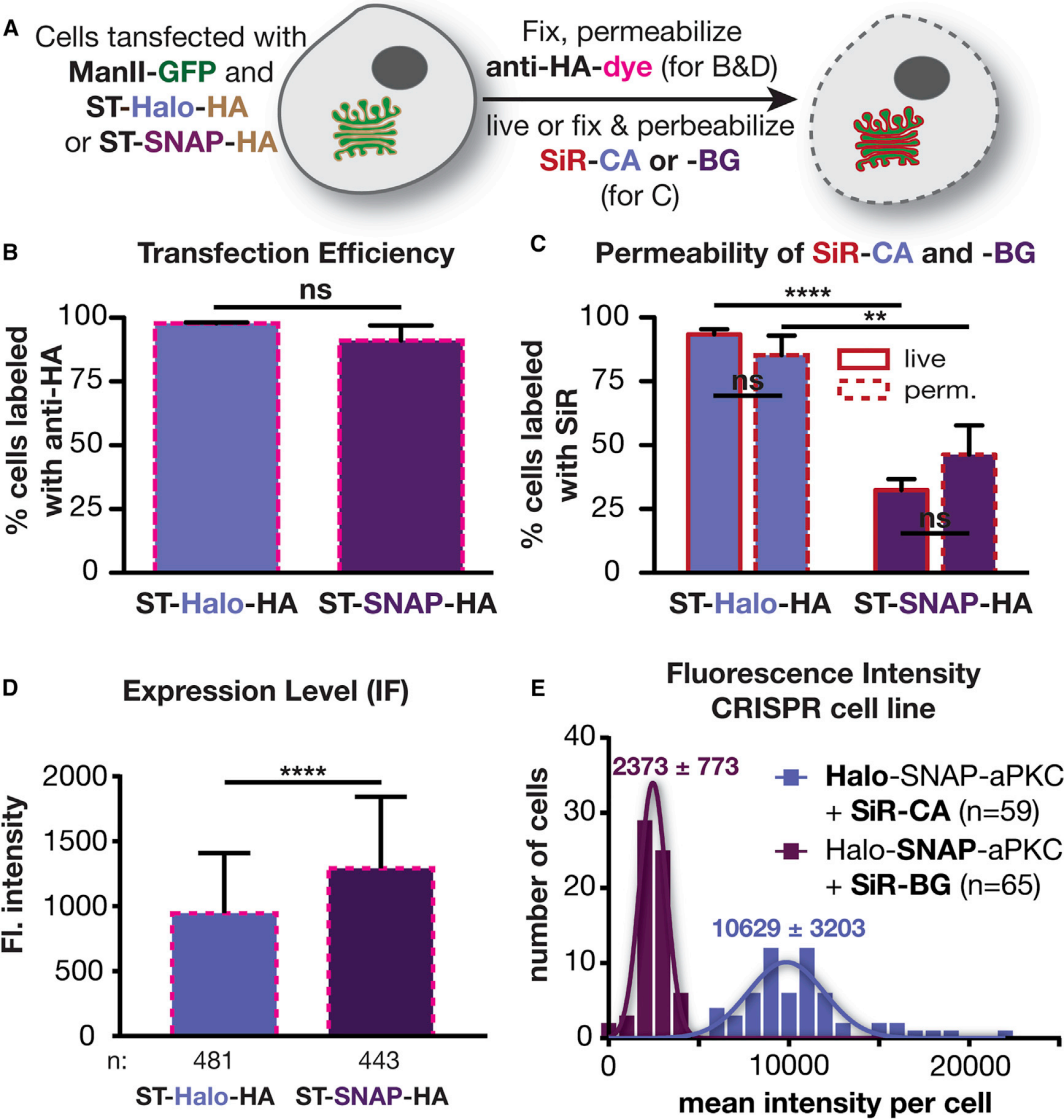
Investigation of Various Factors that Could Cause a Difference in Labeling Using SNAP-tags or HaloTags (A) Scheme of labeling procedures used in (B)–(D). (B) Plot showing the percentage of cells expressing ManII-GFP that have also been immunolabeled with an antibody against the HA tag in three independent experiments (ST-Halo-HA, 463 cells in total; ST-SNAP-HA, 489 cells in total). (C) Comparison of labeling efficiency of live and permeabilized cells using SNAP-tags and HaloTags from three independent experiments (ST-Halo-HA: live, 740 cells in total; fixed and permeabilized, 456 cells in total. ST-SNAP: live, 837 cells in total; fixed and permeabilized, 542 cells in total). (D) Average fluorescence intensity of immunostained cells as described in (A). (E) Intensity distribution in *Drosophila* egg chambers that are expressing Halo-SNAP-aPKC and have been labeled with SiR-CA or SiR-BG.

While potentially interesting, the observed difference between SNAP- and Halo-tagged signals could be due to a number of trivial explanations, including differences in the following: (1) reaction rate between substrate and self-labeling protein, (2) transfection efficiency, (3) cell permeability to the substrates, and (4) expression level of the SNAP and Halo fusion proteins. To exclude the first possibility that the reported difference in reaction rates influenced labeling density, we confirmed that the labeling reaction was complete under the conditions used (Figure S2). To address the other possibilities, we fused a hemagglutinin (HA) tag to the self-labeling proteins as an independent reporter of expression. After labeling with SiR, cells expressing ST-Halo-HA or ST-SNAP-HA were fixed, permeabilized, and incubated with a primary mouse antibody against HA, followed by staining with a secondary goat anti-mouse antibody that was labeled with Alexa 546 (Figure 2A). This allowed us to determine the transfection efficiency independent of SiR labeling. The analysis of the immunolabeled cells showed that 98% of the cells expressing ManII-GFP were positive for ST-Halo-HA, while 91% were positive for ST-SNAP-HA (Figure 2B). Thus, this modest difference in transfection efficiency cannot fully explain the large difference between HaloTag and SNAP-tag labeling.

We next investigated whether differences in cell permeability to the substrates could influence the labeling efficiency. To this end, we tested the labeling of ST-Halo-HA and ST-SNAP-HA in fixed and permeabilized cells—a condition that should negate any potential difference in permeability between SiR-CA and SiR-BG. As shown in Figure 2C, fixation and permeabilization had only a small effect on the labeling efficiency (Figure 2C), indicating that the 3-fold labeling difference seen in the live-cell experiments of Figure 1 is not due to restricted permeability of the SNAP substrate SiR-BG. We note that it is also unlikely that permeability could affect labeling as the reaction was performed with a large excess of substrate (2.5 μM) for 1 h and, as shown in Figure S2, was largely complete under these conditions.

Another trivial explanation for the difference in labeling brightness could be that the expression levels of SNAP and Halo fusion proteins were different. To address this issue, we quantified the fluorescence intensity of the immunolabeling of the HA tag in all cells used for the experiment shown in Figure 2B. Overall, the cells expressing ST-SNAP-HA exhibited a 37% brighter immunofluorescence signal than cells expressing ST-Halo-HA (p > 0.0001), indicating that the SNAP fusion protein is expressed at a slightly higher level than the Halo fusion protein (Figures 2D and S3), contrary to the possibility that SNAP-tag labeling might be dimmer because of a lower expression level.

To further support the above findings, we tagged aPKC endogenously in *Drosophila* using CRISPR/Cas9 technology with homologous recombination to make doubly tagged Halo-SNAP-aPKC flies. aPKC is a kinase that localizes subapically in the follicle epithelium that surrounds the egg chamber (Wodarz et al., 2000). This experimental approach has two important advantages over the experiments described above using mammalian cells: (1) the endogenous protein is tagged and (2) the double tag ensures the same expression levels for Halo and SNAP tags. To investigate the labeling differences in this system, we incubated dissected, fixed ovaries with 600 nM either SiR-CA or SiR-BG to label Halo-SNAP-aPKC. The tissues were imaged under a confocal microscope (Figure S4). Analysis of the images revealed strikingly different mean intensities of egg chambers labeled with SiR-CA and SiR-BG. The mean intensity with SiR-CA was 4.5-fold higher than that with SiR-BG (p < 0.0001) (Figure 2E). This result is in line with the finding in Figure 1C and unequivocally demonstrates that the difference in intensity is not due to different expression levels of SNAP and Halo fusion proteins.

### Brightness of Labeling Depends on Protein of Interest and Dye

**Figure 3:**
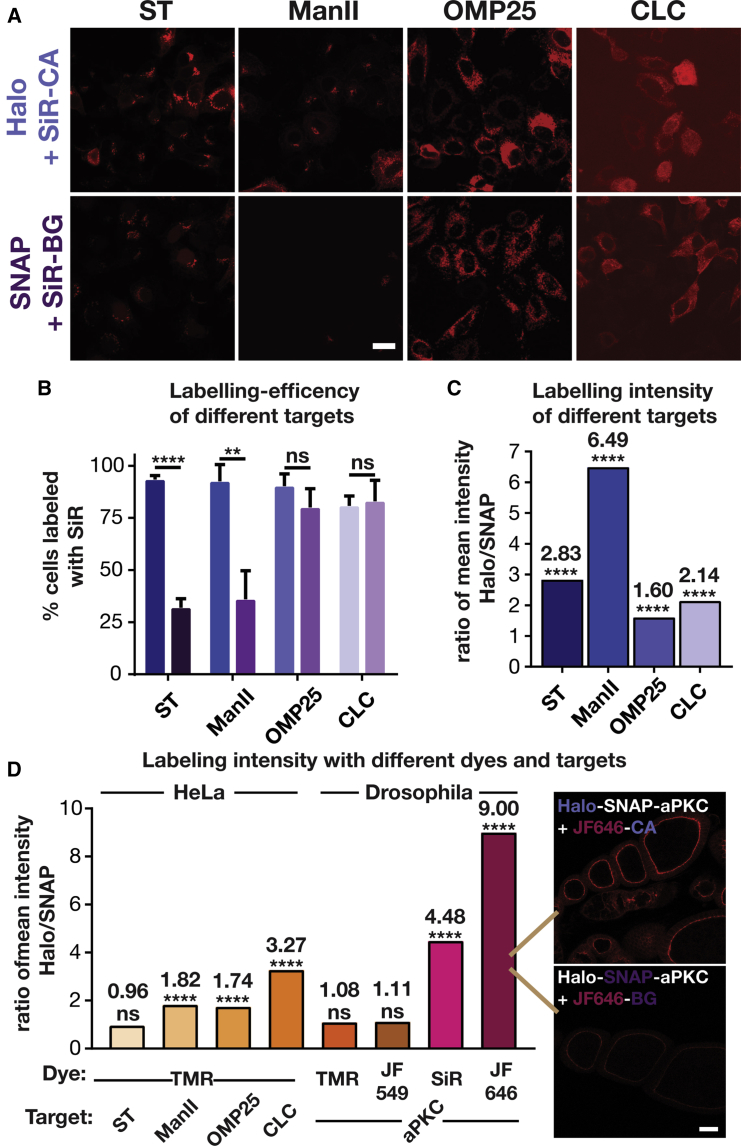
Comparison of HaloTag and SNAP-Tag Labeling with Various Fluorophores of Various Targets in HeLa Cells and *Drosophila* (A) Confocal images of HeLa cells expressing Halo and SNAP fusion proteins of sialyltransferase (ST), mannosidase II (ManII), outer membrane protein 25 (OMP25), and clathrin light chain (CLC) that have been labeled with the corresponding SiR substrates. Scale bar: 20 μm. (B) Labeling efficiency of different targets. The bar graph shows the number of ManII-GFP-expressing cells that were positive for labeling of a fusion protein with SiR from three independent experiments (total number of cells for ST: Halo, 740, SNAP, 837; ManII: Halo, 344, SNAP, 436; OMP25: Halo, 563, SNAP, 524; CLC: Halo, 630, SNAP, 460). Left bars, Halo; Right bars, SNAP. (C) Comparison of the ratio of the mean intensity of various SiR-labeled Halo and SNAP fusion proteins. The intensity distribution for each protein and number of cells analyzed are shown in Figure S4. (D) Comparison of the ratio of the mean intensity of Halo and SNAP fusion proteins labeled with TMR, JF549, SiR, and JF646 in HeLa cells and *Drosophila* egg chambers. The intensity distribution for each protein and number of cells analyzed are shown in Figures S6 and S7. Inset shows the dramatic difference in staining between JF646-CA and JF646-BG in egg chambers. Scale bar: 20 μm.

Since we ruled out the above trivial explanations for the difference between HaloTag and SNAP-tag labeling, we hypothesized that the brightness of the labeling might depend on environmental factors. We, and others, have shown that the fluorescence intensity of carboxyl and hydroxymethyl SiRs correlates with the hydrophobicity of their environment (Erdmann et al., 2014, Lukinavicius et al., 2013, 2014; Takakura et al., 2017, Uno et al., 2014): the more hydrophobic the environment (i.e., the lower its dielectric constant), the less fluorescent the dye. In contrast, methyl SiRs do not show this environmental sensitivity (Koide et al., 2011, Koide et al., 2012). However, since the methyl SiR SNAP substrate led to considerable nonspecific labeling (Figure S5), we did not further investigate this version of the dye. To investigate whether the labeling brightness depends on the protein of interest and its environment, we tested three more fusion proteins in experiments analogous to those of Figure 1. Using HaloTags and SNAP-tags, we labeled ManII, the mitochondrial matrix protein OMP25 (Nemoto and De Camilli, 1999), and the vesicle coat protein clathrin light chain (CLC; Gaidarov et al., 1999) with SiR (Figure 3A). For all proteins tested, the SiR signal was noticeably dimmer in the SNAP-tagged cells. This difference was reflected in both the labeling efficiency (Figure 3B), which is useful but can mask smaller differences, and the labeling intensity of individual cells (Figures 3C and S6). These four pairs of different proteins showed that the extent of the labeling effect can be variable; nevertheless, the general trend was an ∼2- to 6-fold higher labeling intensity with Halo tags. Interestingly, the labeling effect appeared to be greater for transmembrane proteins at the Golgi, potentially due to the local membrane environment. Additional investigation of the photophysical properties of SiR conjugated to Halo and SNAP tags in fluorimetry experiments showed a 3-fold difference in the extinction coefficient between the two conjugates (Table S1). Taking the small difference of the reported quantum yield into account (Lukinavicius et al., 2013), this would represent a 4-fold difference in the brightness of the conjugates, consistent with the difference in labeling brightness observed in cells.

Next, we hypothesized that the dye itself may influence the labeling brightness as different dyes might differentially sense the local environment within HaloTags and SNAP-tags. Thus, we labeled the four SNAP/Halo fusion proteins of ST, ManII, OMP25, and CLC with tetramethylrhodamine (TMR) (Figure S7), which is nearly structurally identical to SiR. TMR substitutes a dimethylsilyl group in SiR with an oxygen, which renders it less electrophilic. As such, TMR is less prone to adopt a nonfluorescent spirolactone, making it less environmentally sensitive (Lukinavicius et al., 2013). Indeed, the difference in brightness of cells with TMR-labeled Halo and SNAP fusion proteins was considerably smaller than the difference with SiR-labeled fusion proteins (Figures 3D and S8). We also tested more rhodamine-based dyes in *Drosophila*, using Halo-SNAP-aPKC. For TMR and its brighter, azetidine-containing analog JF549 (Grimm et al., 2015), we did not observe a significant difference in brightness between Halo and SNAP tags when labeling egg chambers with TMR- or JF549-containing CA or BG substrates, respectively. In stark contrast, we observed a 4.5- to 9-fold difference between SNAP and Halo tags when the same system was labeled with SiR and its azetidine-containing analog JF646 (Grimm et al., 2015), respectively (Figures 3D and S9). As such the JF549/JF646 azetidine dye pair shows the same trend as the TMR/SiR dimethyl rhodamines dye pair, with the far-red dyes showing brighter labeling with the HaloTag under otherwise similar microscopy conditions (see representative image on Figure 3D, right).

We speculate that a combination of several factors might lead to the above observations. The local environment of the tag protein (such as pH) as well as the polarity of its surface can influence the absorption and quantum yield of the dye attached to it. To get a sense of whether the local environments around the dye may differ for HaloTag and SNAP-tag proteins, we surveyed the energy-minimized landscape of SiR tagged to SNAP and Halo proteins, based on the known crystal structures of SNAP (PDB: 3KZZ) and Halo proteins (PDB: 5VNP) (Liu et al., 2017). After energy minimization, we noted the close proximity of the F143 and M174 residues with the SiR dye in the SiR-CA-Halo protein (Figure S9), which might help stabilize the dye in the open state. Finally, intrinsic dye properties, such as the polarity-dependent fluorescence of SiR-based dyes (Erdmann et al., 2014, Lukinavicius et al., 2013), can lead to a different brightness when tagging various self-labeling proteins. Although the contributions of these factors may be multifactorial, our results nevertheless demonstrate that brighter labeling is generally achieved when labeling Halo fusion proteins with SiR dyes.

### Halo/SiR Tagging Is Superior in STED Nanoscopy

Importantly, SiR-based dyes (e.g., SiR and JF646) represent a very important dye class for STED nanoscopy due to their brightness and photostability (Bottanelli et al., 2016, Bottanelli et al., 2017, Erdmann et al., 2014, Lukinavičius et al., 2015, Lukinavičius et al., 2016, Lukinavičius et al., 2014). Near-infrared (IR) dyes avoid cellular green/red autofluorescence, and near-IR light is known to cause much less phototoxicity than green light (Waldchen et al., 2015). Most importantly, SiR dyes, unlike dyes of other classes, are compatible with live-cell super-resolution microscopy since they are cell permeative. Thus, we investigated the difference between SNAP-tag and HaloTag labeling with SiR dyes in the context of STED microscopy.

**Figure 4:**
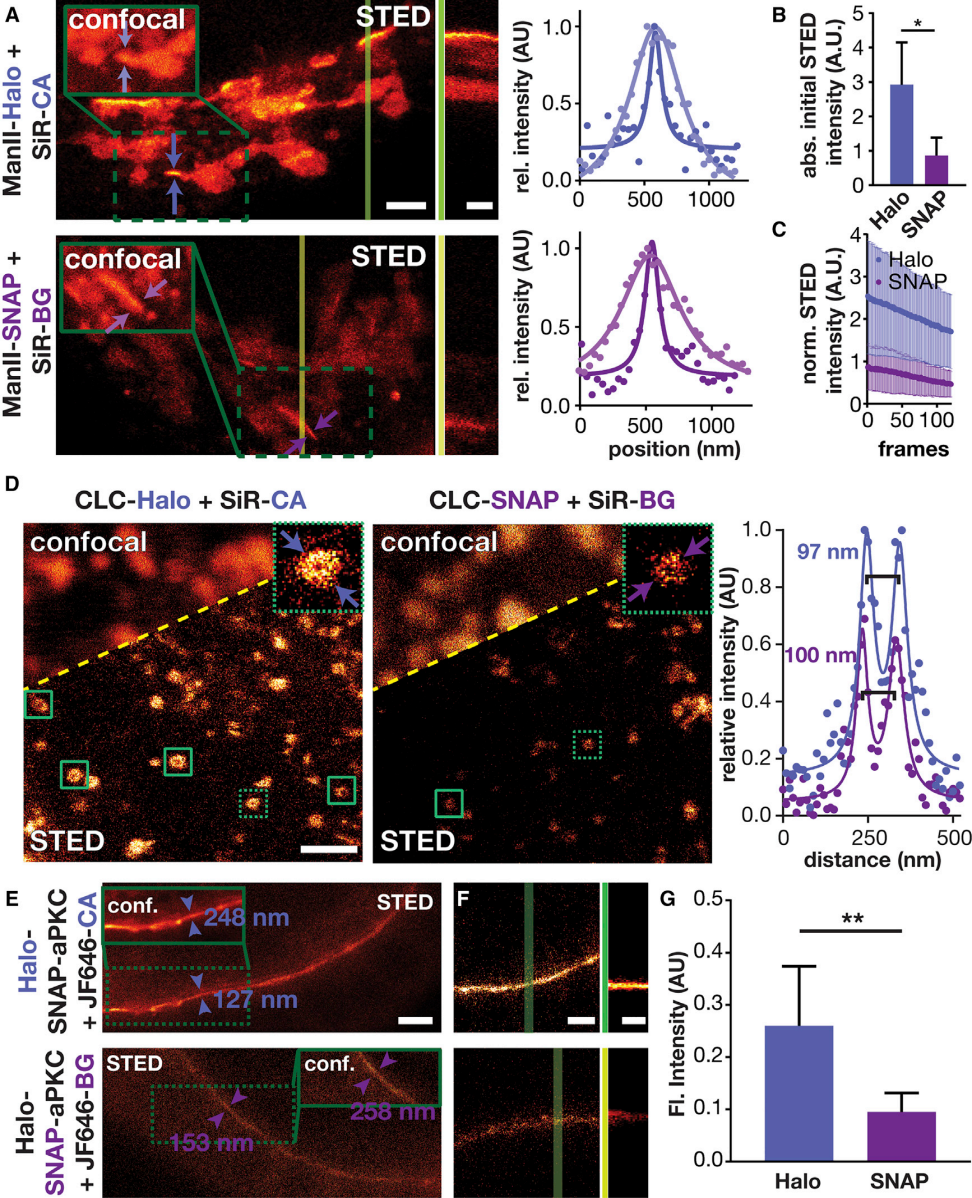
Comparison of Halo and SNAP Tagging in Live-Cell Super-Resolution Imaging (A) STED images of HeLa cells that are transiently expressing ManII-Halo or ManII-SNAP and that have been labeled with SiR-CA or SiR-BG, respectively (scale bar: 2 μm). The insets show the confocal image of the region highlighted with the green box. The vertical dark and light green lines indicate where the kymographs shown in the middle were taken (scale bar: 60 s). The plots show the average fluorescent signal as a function of position between the arrows shown in the confocal and STED images (dots, measured values; lines, fit). (B) Average initial intensity of STED movies of HeLa cells treated as described in (A) (n = 4 cells). (C) Average intensity over time of STED images of HeLa cells treated as described in (A) (n = 4 cells). (D) STED images of HeLa cells that are expressing Halo-CLC or SNAP-CLC and were labeled with SiR-CA or SiR-BG, respectively. The green boxes highlight clathrin-coated pits with a hollow center. Magnifications of the clathrin-coated pits highlighted with the dashed green boxes are shown in the upper right corner (scale bar: 1 μm). The plots show the average fluorescent signal as a function of position between the arrows shown in the STED images (dots, measured values; lines, fit). (E) STED images of *Drosophila* egg chambers expressing Halo-SNAP-aPKC that have been labeled with JF646-CA (top) or JF-646-BG (bottom). The insets show a confocal image of the area in the green dashed box. The values indicate the full-width half-maximum of line profiles taken between the arrowheads. Scale bar: 2 μm. (F) First frame of an STED video of *Drosophila* egg chambers that have been treated as described in (E). Scale bar: 1 μm. The green lines indicate where the kymographs shown next to it have been taken. Scale bar: 100 s. (G) Average initial intensity of STED movies of *Drosophila* egg chambers that have been treated as described in (E) (Halo, n = 4; SNAP, n = 6).

First, we imaged the Golgi in HeLa cells transiently expressing ManII-Halo and ManII-SNAP, both labeled with SiR, in confocal and STED mode (Figure 4A and Video S1). As expected, we observed an improvement in resolution in the STED mode compared with the confocal mode. Strikingly, the initial brightness of the Halo-labeled proteins was about 3-fold brighter than that of SNAP-labeled proteins (Figure 4B). The STED kymographs (Figure 4A) and bleaching profile (Figure 4C) show that only the sample labeled using HaloTag was bright for over 100 s. These findings are consistent with a recent single-molecule tracking study, which reported that HaloTag conjugates are more photostable than SNAP-tag conjugates (Presman et al., 2017). As a second example, we imaged CLC, which labels clathrin-coated endocytic pits. Showing the power of STED, clathrin-coated pits appeared as blurry spots in confocal images, but appeared as donuts in STED images, with the expected diameter of approximately 100 nm (Figure 4D) (Huang et al., 2013). Similar to the Golgi labeling, the HaloTag-labeled clathrin structures exhibited a brighter fluorescent signal (Figure S8) and more of them showed a clearly resolved hollow center, compared with SNAP-tagged structures. These observations demonstrate that the tags differentially affected STED image quality.

Video S1. Comparison of STED Videos of HeLa Cells Transiently Expressing ManII-Halo or ManII-SNAP that Were Labeled with SiR-HaloTag ligand (SiR-CA) or SiR-SNAP-tag Ligand (SiR-BG), Respectively, Related to Figure 4

Third, we imaged Halo-SNAP-aPKC labeled with JF646 in live *Drosophila* egg chambers by STED microscopy. As expected, the STED images exhibited 2-fold higher resolution than the confocal images, independent of the tagging system. However, the aPKC labeled with JF646-CA was 2.5-fold brighter than the aPKC labeled with JF646-BG (Figure 4F). The difference in brightness also had a significant impact on the time span over which the signal could be observed. As shown in the kymographs, egg chambers in which Halo-SNAP-aPKC was labeled with JF646-CA still showed a bright signal after 170 s, whereas the signal in egg chambers labeled with JF646-BG was hardly distinguishable from background signal at this period (Figure 4G and Video S2).

Video S2. Comparison of STED Videos of *Drosophila* Egg Chambers Expressing Halo-SNAP-aPKC that Were Labeled with SiR-HaloTag Ligand (SiR-CA) or SiR-SNAP-tag Ligand (SiR-BG), Respectively, Related to Figure 4 In the top half of the video the contrast/intensity settings are optimized to display the Halo-labeled cell, whereas the settings are optimized to display the SNAP-labeled cell in the bottom.

Together these three different examples strongly argue that Halo tagging is superior to SNAP tagging for live-cell STED imaging when using SiR-based dyes. Halo proteins tagged with SiR give a brighter signal, which leads to higher quality images and allows the acquisition of more images.

### Guidelines for the Usage of Self-Labeling Proteins for Imaging Applications

For single-color imaging of SiR-based dyes, by either confocal or STED microscopy, we recommend Halo tagging as a first choice, since it provides fluorescence that is brighter and less prone to bleaching rapidly. For two-color imaging, both the brightness and the environmental sensitivity of the dyes need to be taken into account in deciding which dye should be paired with which self-labeling protein. As Halo- and SNAP-tagging strategies are orthogonal, a reasonable strategy would be to use the less bright and/or more environmentally sensitive dye with Halo tagging; the brighter and less environmentally sensitive dye should be used with SNAP tagging (for examples, see Bottanelli et al., 2017, Bottanelli et al., 2016). Similar considerations can be extrapolated to single-molecule-switching microscopy modalities (also termed PALM or STORM), as the brightness of a label in this super-resolution technique directly correlates with the localization precision/resolution.

## Significance

**Self-labeling proteins are the method of choice for covalently attaching dyes to proteins for imaging applications that demand bright and photostable fluorophores. In particular, STED microscopy, but also other super-resolution methods, and long time-lapse 3D microscopy heavily rely on self-labeling proteins. In our study, we systematically compared two self-labeling proteins, SNAP-tags and HaloTags, with respect to expression levels, substrate permeability, target protein, and dye used for labeling. The results show that when using silicone rhodamine derivatives, Halo tagging is far superior to SNAP tagging, resulting in typically ∼4-fold brighter structures and correspondingly longer live-cell STED movies. The differences shown here are dependent on both the protein of interest and the labeling dye. We further suggest dual-labeling strategy guidelines to help avoid testing of all combinations of dyes and self-labeling proteins.**

## STAR★Methods

### Key Resources Table

**Table:** 

REAGENT or RESOURCE	SOURCE	IDENTIFIER
**Chemicals, Peptides, and Recombinant Proteins**		

SiR-HaloTag ligand (SiR-CA)	Promega	N/A (gift)
SiR-SNAP-tag ligand (SiR-BG)	Lukinavicius et al., 2013	N/A (gift)
TMR-HaloTag ligand (TMR-CA)	Promega	Cat# G8251
TMR-SNAP-tag ligand /SNAP-Cell® TMR-Star (TMR-CP)	NEB	Cat# S9105S
JF646-HaloTag ligand (JF646-CA)	Grimm et al., 2015	N/A
JF646-SNAP-tag ligand (JF646-BG)	Grimm et al., 2015	N/A
JF549-HaloTag ligand (JF549-CA)	Grimm et al., 2015	N/A
JF549-SNAP-tag ligand (JF549-BG)	Grimm et al., 2015	N/A
MeSiR-HaloTag ligand (MeSiR-CA)	Lukinavicius et al., 2013	N/A
MeSiR-SNAP-tag ligand (MeSiR-BG)	Lukinavicius et al., 2013	N/A

**Experimental Models: Cell Lines**		

HeLa	ATCC	ATCC CCL-2

**Experimental Models: Organisms/Strains**		

Cas9 expressing fly line CFD2	Port et al., 2015	N/A

**Oligonucleotides**		

Primers and gBlock Used for Site-Directed Mutagenesis	This paper	N/A

**Software and Algorithms**		

Prism Graphpad	Graphpad	Graphpad.com
Pymol	Schrödinger	https://www.schrodinger.com/pymol
UCSF Chimera	Pettersen et al., 2004	N/A
YASARA	Krieger and Vriend , 2015	N/A

### Contact for Reagent and Resource Sharing

Further information and requests for resources and reagents should be directed to and will be fulfilled by the Lead Contact, Derek Toomre (derek.toomre@yale.edu).

### Experimental Model and Subject Details

#### Cell Culture

HeLa cells were cultured in Dulbecco's Modified Eagle Medium (DMEM) (Gibco) supplemented with 10% fetal bovine serum (FBS) (Sigma-Aldrich), penicillin (100 unit/mL) and streptomycin (100 μg/mL). The cells were cultured at 37°C in a CO_2_/air (5%/95%) incubator. The sex of these cell lines is female.

#### Drosophila

Cas9 expressing fly line CFD2 in which the Cas9 protein is expressed from the *nanos* promoter was described earlier (Port et al., 2015).

### Method Details

#### Fly Stock

The aPKC gene was tagged using the CRISPR/Cas9 system. A CRISPR target 60bp 3’ of the initiating methionine codon (GAATAGCGCCAGTATGAACATGG) was targeted using an *in vitro* transcribed guide RNA prepared as described by Bassett (Bassett et al., 2013). The sgRNA was co-injected with a homologous recombination donor plasmid – pCRII/HASP-aPKC in which a Halo-SNAP double tag was flanked by 1.2 and 2.0kb left and right homology arms from the aPKC gene inserted into pCRII TOPO cloning vector (Invitrogen). The CRISPR site was modified in the donor to prevent cleavage of the donor. Guide and donor were injected into embryos (80ng/ul and 300ng/ul respectively) from the Cas9 expressing fly line CFD2 (Port et al., 2015) in which the Cas9 protein is expressed from the *nanos* promoter.

Single adult flies from the injection were mated to *yw* flies and were left to produce larvae, after which the adult was recovered for analysis. PCR analysis of the F^0^ parent, using primers specific for the insertion of the HaSP tag, was carried out to identify individuals most likely to yield insertions. 10-12 F^1^ progeny from these flies were then singly mated to the appropriate balancer stock, following which the F^1^ parent was sacrificed to confirm insertion of the HASP tag. This resulted in four separate lines of HaSP-aPKC flies derived from two separate F^0^ individuals.

#### Plasmids

##### ManII-GFP

ManII-GFP (Lavieu et al., 2013) was used in a previous study.

##### ST-SNAP-HA and ST-Halo-HA

ST was amplified from ST-RFP (a gift from the Roher lab) as a 5’EcoRI-3’XbaI PCR fragment and cloned into an EcoRI-XbaI digested pC4S1 plasmid (Takara Bio Inc). The HA tag sequence is part of the pC4S1 plasmid (following the SpeI site).

##### ManII-SNAP and ManII-Halo

Myc-SNAP was amplified from pSNAP_*f*_ (NEB) as a 5’XbaI-3’SpeI PCR fragment and cloned into the XbaI-SpeI digested ManII-Halo (Bottanelli et al., 2016).

##### SNAP-OMP25 and Halo-OMP25

Halo was amplified from the pFN23K-Halo plasmid (Promega, G2861) as a 5’XbaI-3’SpeI PCR fragment and cloned into a XbaI-SpeI digested SNAP-OMP25 (Bottanelli et al., 2016) plasmid.

##### SNAP-CLC and Halo-CLC

Clatharin Light Chain (CLC) was vector obtained from the Bewersdorf lab (Huang et al., 2013)SNAP and Halo were amplified and placed in the CLC vector using Age1 and Xho1.

#### Transfection

##### For Confocal Imaging

Hela cells were seeded in 24 well plates with glass coverslips the day before transfection. Cells were transfected with 0.5 μg (4 μg for CLC) of either the SNAP or Halo fusion protein encoding plasmid and 0.25 μg of the ManII-GFP encoding plasmid using 2 μL Lipofectamine 2000 (life technologies) per well following the manufacturer’s protocol.

##### For STED Imaging

Cells were seeded in 35 mm glass bottom dishes (Mattek P35G-1.5-14-C). Cells were transfected with plasmids encoding ST-SNAP-HA or ST-Halo-HA (∼1 μg), ManIIGFP (∼0.5 μg) and 10 μL of Lipofectamine 2000. Alternatively, cells were transfected with 8 μg of the plasmids encoding SNAP-CLC or Halo-CLC.

#### Live Cell Labeling (Hela)

Cells were seeded in 35 mm glass bottom dishes. Cells were transfected with plasmids encoding Halo or SNAP tag fusion proteins (∼1 μg) as well as ManIIGFP (∼0.5 μg) and 10 μL of Lipofectamine (2000). One day after transfection, cells were incubated with 2.5 μM of dye-substrate respectively in DMEM for 1 h at 37°C. Subsequently, the cells were washed three times with DMEM and placed back in the incubator for a 2 hour washout of the dye before imaging.

Cells transiently expressing either a Halo or SNAP-tag fusion protein were incubated with 2.5 μM of SiR-HaloTag-ligand (a gift from Promega) or SiR-SNAP-tag ligand (a gift from Kai Johnsson, EPFL), respectively in DMEM for 1 h at 37°C. For STED experiments, 5 μM solutions of the ligands were used. For labeling with TMR, cells were incubated for 30 mins with 100 nM of HaloTag^®^ TMR Ligand (a gift from Promega) or SNAP-Cell^®^ TMR-Star (NEB). Subsequently, the cells were washed three times with DMEM and placed back in the incubator for a 2 hour washout of the dye before imaging.

Note: SNAP-Cell^®^ TMR-Star is a chloropyrimidine substrate for SNAP tag that has been shown to perform better than TMR-BG (Ivan et al., 2013).

#### Egg Chamber Labeling

Ovaries (female flies) were dissected in PBS and fixed in 4% PFA (in PBS) for 30 min at room temperature. Samples were washed 3x 5 min in PBS. For SiR/TMR comparisons samples were stained with either Halo-SiR and SNAP-TMR, or Halo-TMR and SNAP-SiR, all diluted to 0.6 μM in PBS. For JF549/JF646 comparisons samples were stained with either Halo-JF646 and SNAP-JF549, or Halo-JF549 and SNAP-JF646, diluted to 0.5 μM in PBS (Grimm et al., 2015). Samples were stained at 37°C with shaking for 30 min. Samples were then washed 6x 10 min in PBT (0.1% Triton X-100) and mounted in Vectashield with DAPI.

#### Cell Fixation

Cells were washed 3 times with PBS (American Bioanalytical) and fixed with 4% PFA (Electron Microscopy Sciences) for 20 minutes.

#### Concentration Dependent Labeling Studies

Hela cells were transfected with ST-Halo-HA or ST-SNAP-HA and incubated with different concentrations of SiR substrates at 37°C for 1 h. Subsequently, the cells were washed 3 times with medium and incubated with fresh medium for 2 h at 37°C. The cells were fixed as described above and imaged using a confocal microscope.

#### Pulse Chase Studies

Hela cells were transfected with ST-Halo-HA or ST-SNAP-HA and incubated with different concentrations of SiR substrates at 37°C for 30 min. The cells were washed once and 2.5 μM of TMR substrate was added and the cells were incubated for 30 min at 37°C. Subsequently, the cells were washed 3 times with medium and incubated with fresh medium for 2 h at 37°C. The cells were fixed as described above and imaged using a confocal microscope.

#### Immunolabeling

After fixation the cells were permeabilized with 0.1% Triton X (American Bioanalytical) for 10 minutes. They were washed 3 times with PBS. The cells were blocked with 3% IgG free BSA (Accurate Chemicals). The primary HA antibody (Covance) was diluted 1:1000 in 3% BSA. Subsequently, the cells were incubated with this solution for 1 h at room temperature. The cells were then washed 3 times with 3% BSA and labeled with the secondary antibody for 30 minutes at room temperature. Depending on the original live cell staining, the secondary antibody was either Alexa 546 goat anti-mouse (Halo/SNAP-tag labeling with SiR) or Alexa 647 goat anti-mouse (Halo/SNAP-tag labeling with TMR) (Invitrogen). The secondary antibody was diluted 1:1000 in 3% BSA. Subsequently the cells were washed 3 times with PBS and once with water. Next, they were mounted onto glass microscopes slides with ProLong® Gold Antifade (Life Technologies). The slides were protected from light overnight at room temperature before imaging.

#### SNAP and Halo-tag Labeling after Fixation and Permeabilization

Cells transiently expressing either a Halo or SNAP tag fusion protein were fixed as described above and permeabilized with 0.1% TritonX for 5 min. Subsequently they were labeled in analogy to the live cell with the exception that the substrates were dissolved in 3% BSA. After washing three times with DMEM the cells were mounted as described above.

#### Confocal Imaging of Labeled Hela Cells

Images were taken on a Zeiss 510 Confocal, using a 63x 1.4 Oil DIC objective. GFP was excited with a 488 nm laser at a 35% intensity and detected after a 505-530 band pass filter and a pinhole set to 98 μm. TMR and Alexa 546 were exited with a 543 nm laser at a 30% intensity and detected after a 560-615 band pass filter and 100 μm pinhole. SiR and Alexa 647 were excited with a 633 nm laser at an 11% intensity and detected after a 650 nm long pass filter and a pinhole set to100 μm.

#### Confocal Imaging of Egg Chambers

Images were taken on a Leica SP8 Confocal, using a 63x 1.4 Oil objective. The tunable filters were set up with singly stained samples to ensure that no signal from the TMR/JF549 channel could bleed through to the SiR/JF646 channel or vice versa. Laser levels were set to ensure no pixels were saturated to enable accurate signal quantification, and all images using the same fluorophores were obtained with identical acquisition settings.

#### STED Microscopy of Labeled Hela Cells

STED imaging was performed on a custom built system (Bewersdorf lab, Yale University) centered around an 80 MHz mode-locked Ti:Sapphhire laser (Chameleon Ultra II, Coherent) tuned to 755 nm as the STED depletion beam (For more details on the instrument see Supplemental Information of an earlier publication(Erdmann et al., 2014)). Imaging of SiR labeled ManII (Figure 4A) was achieved with 640 nm excitation, 40 nm pixel size, a 512 by 512 image format, 775 nm STED laser, and 32 accumulations per line resulting in a frame rate of 0.98 fps. Imaging of SiR labeled CLC (Figure 4D) was achieved with 640 nm excitation, 10 nm pixel size, a 1024 by 1024 image format, 755 nm STED laser, and 120 accumulations per line resulting in a frame rate of 0.26 fps.

#### STED Microscopy of Labeled Egg Chambers

Ovaries were dissected in Express Five + Glutamate medium (Life Technologies), supplemented with 10 μg/ml insulin (I9278, Sigma) containing 1 μM dye. After staining (15 min for SiR, 20 min for JF646), dissected ovarioles were transferred to Express Five containing no dye, to wash for 30 min. Ovarioles were transferred to Poly-L-Lysine coated 8-well μ-Slides (80824, Ibidi) with ∼200 μl medium. Ovarioles were imaged on a custom build STED microscope similar to the one described above, with a 100x Oil objective, STED laser (775 nm) power of ∼120 mW and a 640 nm laser for sample excitation. The 512 by 512 images were recorded with 60 line accumulations and a pixel size of 19.53 nm, resulting in a frame rate of 0.52 fps.

#### STED Image Processing

For improved presentation in Figure 4 as well as Videos S1 and S2 the raw microscopy data were Gaussian blurred (0.5 pixels) in ImageJ. For Figure 4A and Video S1, four recorded frames were summed to obtain one image or one frame of the movie, respectively (3.91 sec/frame). For Video S2 as well ass Figures 4E and 4F, five frames were summed to obtain one image or one frame of the Video (9.53 sec/frame).

#### Image Analysis (Hela Cells)

##### Labeling Efficiency

All cells positive for ManII-GFP were identified and if the same cell was also positive for labeling of a fusion protein (Golgi-like structure visible) it was counted as labeled (Figures 1A, 2C, and 3B).

##### Labeling Intensity

The mean intensity of each labeled area (Golgi, mitrochondria, clathrincoated pit) was subtracted from the mean intensity of an unlabeled area (background) using Image J. Over 270 cells were analyzed from at least three independent experiments for each condition (Figures 1C, 3C, and 3D).

##### Transfection Efficiency

All cells positive for ManII-GFP were identified and if the same cell was also immunolabeled with an anti-HA antibody it was counted as successfully transfected (Figure 2B).

##### Expression Level

The mean intensity of each immunolabeled Golgi was subtracted from the mean intensity of an unlabeled area (background) using Image J (Figure 2D).

#### Image Analysis (*Drosophila* Egg Chambers)

##### Labeling Intensity

At least five experiments were performed to measure the SNAP and Halo staining for each dye, with between 7 and 14 images analyzed for each experiment (Figure 3D). We created a custom plugin (available on request) for ICY (de Chaumont et al., 2012) to perform semi-automated quantification of aPKC labelling of egg chambers in whole images or user-defined regions. The signal area was mapped using a threshold calculated by applying the Renyi entropy method (Kapur et al., 1985) to a difference of Gaussians processed copy of the image (sigma=4, k=1.4) and extracting the composite of all regions with a minimum area of 500 px (16.24 μm^2^). The mean intensity of the mapped line is measured as signal, and the area outside of the line is measured as background. Measurements were obtained for signal and background for both the TMR (or JF549) and SiR (or JF646) channels and recorded in Excel. Background corrected measurements were recorded in GraphPad Prism for further analysis.

#### Production of Plasmids Encoding SNAP-tag-His_6_ and Halo-tag-His_6_

For these experiments, we prepared SNAP and Halo-tag constructs bearing a C-terminal His_6_ tag for overexpression and purification from E. coli. A plasmid encoding SNAP-tag-His_6_ was prepared using Gibson assembly. A gBlock encoding SNAP-tag-His_6_ (SNAP26b) was purchased from Integrated DNA Technologies. SNAP-tag-His_6_ was inserted into a linearized pET vector (pET32A, Novagen) using Gibson Assembly® MasterMix (NEB) in accordance with the manufacturer’s protocol. His_6_HaloTag® T7 Vector was purchased from Promega. The His_6_HaloTag® T7 Vector was modified to encode Halo-tag-His_6_ in two rounds of mutagenesis. First, the N-terminal His6 tag was excised from the vector. Next, a His_6_ tag, followed by a stop codon, was inserted into the C-terminus of Halo-tag by site-directed mutagenesis.

#### Primers Used for Site-Directed Mutagenesis

##### Excision of N-terminal His_6_ tag from HaloTag® T7

5′-CATGATGAATTCTCCTTAGTAAAG-3′

5′-GCAGAAATCGGTACTGGC-3′

##### Insertion of C-terminal His_6_ tag into HaloTag® T7

5′-CTAATGGTGATGGTGATGGTGGCCGGAAATCTCGAGCGTC-3′

5′-GAGCCAACCACTGAGGATC-3′

##### Linearization of PET32A for Gibson Assembly

5′-ATGTATATCTCCTTCTTAAAGTTAAACAAAATTATT-3′

5′-TAACAAAGCCCGAAAGGAAG-3′

##### gBlock Encoding SNAP-tag-His_6_

The requisite overhangs for the Gibson assembly reaction are shown in lowercase.

tttaagaaggagatatacatATGGATAAAGATTGTGAGATGAAGCGCACCACACTTGACTCACCGCTGGGGAAACTTGAATTGTCGGGATGCGAGCAAGGTTTGCATGAGATTAAGCTGTTAGGTAAAGGAACATCTGCCGCAGACGCCGTCGAAGTTCCTGCCCCGGCTGCGGTCTTAGGGGGTCCAGAGCCCCTTATGCAGGCGACTGCCTGGCTTAATGCCTACTTCCACCAACCAGAAGCCATCGAGGAATTTCCGGTTCCGGCACTGCACCACCCTGTTTTCCAACAAGAGAGCTTCACACGTCAGGTGTTGTGGAAGCTGTTAAAAGTTGTTAAATTTGGAGAGGTCATCTCATACCAACAGTTAGCCGCACTGGCCGGTAATCCGGCGGCAACAGCAGCCGTCAAAACAGCCCTGAGTGGTAATCCAGTACCTATCTTAATCCCCTGCCATCGCGTTGTGAGTTCGAGCGGTGCAGTCGGCGGTTATGAAGGAGGTTTAGCAGTGAAGGAGTGGTTACTGGCCCATGAGGGTCATCGTCTGGGGAAGCCGGGCTTAGGTCATCACCATCACCACCACtaacaaagcccgaaagg.

#### Expressed Protein Sequences

##### Halo-tag-His_6_

MAEIGTGFPFDPHYVEVLGERMHYVDVGPRDGTPVLFLHGNPTSSYVWRNIIPHVAPTHRCIAPDLIGMGKSDKPDLGYFFDDHVRFMDAFIEALGLEEVVLVIHDWGSALGFHWAKRNPERVKGIAFMEFIRPIPTWDEWPEFARETFQAFRTTDVGRKLIIDQNVFIEGTLPMGVVRPLTEVEMDHYREPFLNPVDREPLWRFPNELPIAGEPANIVALVEEYMDWLHQSPVPKLLFWGTPGVLIPPAEAARLAKSLPNCKAVDIGPGLNLLQEDNPDLIGSEIARWLSTLEISGHHHHHH

##### SNAP-tag-His_6_

MDKDCEMKRTTLDSPLGKLELSGCEQGLHEIKLLGKGTSAADAVEVPAPAAVLGGPEPLMQATAWLNAYFHQPEAIEEFPVPALHHPVFQQESFTRQVLWKLLKVVKFGEVISYQQLAALAGNPAATAAVKTALSGNPVPILIPCHRVVSSSGAVGGYEGGLAVKEWLLAHEGH RLGKPGLGHHHHHH.

#### Overexpression of Halo-tag-His_6_ and SNAP-tag-His_6_

Plasmids encoding Halo-tag-His_6_ and SNAP-tag-His_6_ were transformed into BL21(DE3) pLysS Competent Cells (Agilent Technologies). Single colonies were used to inoculate 5 mL of LB medium supplemented with ampicillin (100 μg/mL). The cultures were grown at 37°C with shaking at 220 rpm. The primary cultures were used to inoculate 1 L of LB medium supplemented with ampicillin. The secondary culture was grown at 37°C until the OD_600_ reached 0.6. The secondary culture was then cooled to 18°C and protein expression was induced by the addition of IPTG (238 mgs, final concentration 1 mM). After 12 hrs, the cells were harvested by centrifugation and lysed in 20 mL of 20 mM Tris pH 8.0, 150 mM NaCl, and 1 mM DTT supplemented with one protease inhibitor tablet (cOmplete, Mini, EDTA-free Protease Inhibitor Cocktail, Sigma). After clearing the lysate by centrifugation, 2 mL of TALON® resin was added to the lysate and incubated for 1 hr at 4°C. The resin was then transferred to a disposable column and washed with 2 X 20 mM Tris pH 8.0, 150 mM NaCl followed by a wash with 2 X 20 mM Tris pH 8.0, 1 M NaCl, 1 mM DTT and 10 mM imidazole. Next, the proteins were eluted from the resin in 1 mL aliquots with 20 mM Tris pH 8.0, 150 mM NaCl, 1 mM DTT containing 250 mM imidazole. Elution fractions were analyzed by SDS-PAGE, and the cleanest fractions were pooled and dialyzed overnight into 20 mM Tris pH 8.0, 150 mM NaCl, containing 1 mM DTT at 4°C. Finally, the concentration of Halo-tag-His_6_ and SNAP-tag-His_6_ was determined with the Pierce™ 660 nm Protein Assay Reagent using bovine serum albumin (BSA) as a standard.

#### SDS-PAGE Analysis of Halo-tag-His_6_ and SNAP-tag-His_6_

**Figure:**
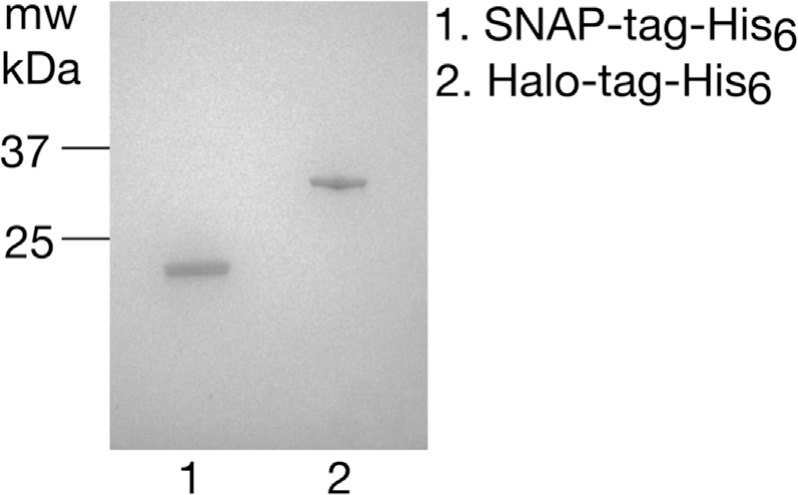

#### Mass Spectrometry Analysis of SNAP-tag-His_6_ and Halo-tag-His_6_

**Figure:**
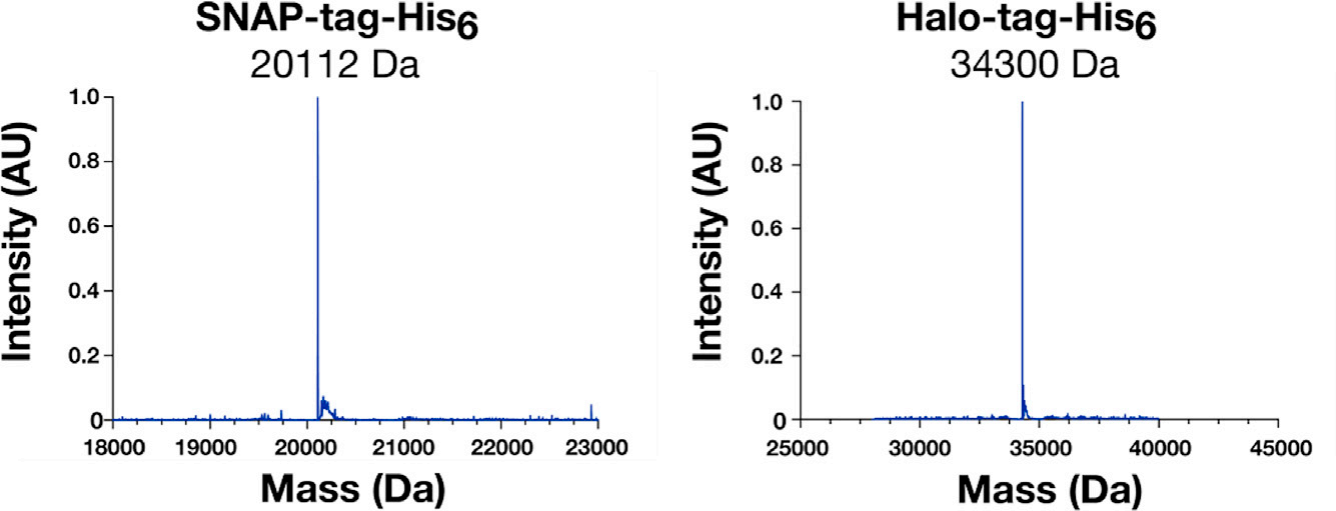

#### Absorbance Experiments

First, we measured the concentration of each concentrated stock solution (BG-SiR and CA-SiR) by diluting an aliquot of each dye into enzyme buffer (20 mM Tris pH 8.0, 150 mM NaCl, 1 mM DTT) containing 0.1% SDS. The concentration of each stock solution was determined using a previously reported extinction coefficient(Lukinavicius et al., 2013) (100,000 M^-1^ cm^-1^) corresponding to each dye dissolved in 0.1% SDS. To estimate the extinction coefficient of each dye in the absence of enzyme or SDS, we prepared 10 μM solutions of each dye in buffer. In the absence of enzyme or SDS, we measured an extinction coefficient of 10,100 M^-1^ cm^-1^ and 8,300 M^-1^ cm^-1^ for SiR-BG and SiR-CA, respectively. To estimate the extinction coefficient of SNAP-SiR and Halo-SiR, we prepared equal volume solutions containing 10 μM of each dye in the presence of 3 molar equivalents (30 μM) of each enzyme, incubated the solutions for 1.5 hrs at 37°C, and measured the resulting absorbance, which should correspond to a 10 μM solution of labeled protein. We determined an extinction coefficient of 43’200 M^−1^ cm^−1^ for SNAP-SiR and 130,200 M^−1^ cm^−1^ for Halo-SiR. To ensure that all of the free dye was consumed in the reaction, we repeated the experiment in the presence of 6 molar equivalents (60 μM) of each enzyme and measured the resulting absorbance. The increase in absorbance was the same whether 3 or 6 molar equivalents of enzyme was added in each case, demonstrating that all of the dye was consumed in the presence of excess protein.

#### Energy Minimization

The fused molecules of SiR-CA and SiR-BG were generated by UCSF Chimera (Pettersen et al., 2004), and the figure was prepared with PyMol (Schrödinger). Energy minimization were performed using YASARA (Krieger and Vriend, 2015).

### Quantification and Statistical Analysis

Statistical significance was determined using two-tailed unpaired t-tests in Prism Graphpad. n-values are indicated in text or figure legends. P-values were indicated as follows: ns: p > 0.05, *: p < 0.05, **: p < 0.01, ***: p < 0.001, ****: p < 0.0001. If not indicated otherwise, data are shown as means ± SD of three or more independent experiments.

## Supplemental Information

Document S1. Figures S1–S9 and Table S1

Document S2. Article plus Supplemental Information

## References

[bib39] Bassett Andrew R., Tibbit C., Ponting Chris P., Liu J.-L. (2013). Highly efficient targeted mutagenesis of Drosophila with the CRISPR/Cas9 system. Cell Rep..

[bib1] Bottanelli F., Kilian N., Ernst A.M., Rivera-Molina F., Schroeder L.K., Kromann E.B., Lessard M.D., Erdmann R.S., Schepartz A., Baddeley D. (2017). A novel physiological role for ARF1 in the formation of bi-directional tubules from the Golgi. Mol. Biol. Cell.

[bib2] Bottanelli F., Kromann E.B., Allgeyer E.S., Erdmann R.S., Wood Baguley S., Sirinakis G., Schepartz A., Baddeley D., Toomre D.K., Rothman J.E. (2016). Two-colour live-cell nanoscale imaging of intracellular targets. Nat. Commun..

[bib40] de Chaumont F., Dallongeville S., Chenouard N., Herve N., Pop S., Provoost T., Meas-Yedid V., Pankajakshan P., Lecomte T., Le Montagner Y. (2012). Icy: an open bioimage informatics platform for extended reproducible research. Nat. Meth..

[bib3] Dempsey G.T., Vaughan J.C., Chen K.H., Bates M., Zhuang X.W. (2011). Evaluation of fluorophores for optimal performance in localization-based super-resolution imaging. Nat. Methods.

[bib4] Erdmann R.S., Takakura H., Thompson A.D., Rivera-Molina F., Allgeyer E.S., Bewersdorf J., Toomre D., Schepartz A. (2014). Super-resolution imaging of the golgi in live cells with a bioorthogonal ceramide probe. Angew. Chem. Int. Ed..

[bib5] Fernandez-Suarez M., Ting A.Y. (2008). Fluorescent probes for super-resolution imaging in living cells. Nat. Rev. Mol. Cell Biol..

[bib6] Fornasiero E.F., Opazo F. (2015). Super-resolution imaging for cell biologists. BioEssays.

[bib7] Gaidarov I., Santini F., Warren R.A., Keen J.H. (1999). Spatial control of coated-pit dynamics in living cells. Nat. Cell Biol..

[bib8] Gautier A., Juillerat A., Heinis C., Correa I.R., Kindermann M., Beaufils F., Johnsson K. (2008). An engineered protein tag for multiprotein labeling in living cells. Chem. Biol..

[bib9] Grimm J.B., English B.P., Chen J., Slaughter J.P., Zhang Z., Revyakin A., Patel R., Macklin J.J., Normanno D., Singer R.H. (2015). A general method to improve fluorophores for live-cell and single-molecule microscopy. Nat. Methods.

[bib10] Hell S.W. (2007). Far-field optical nanoscopy. Science.

[bib11] Hinner M.J., Johnsson K. (2010). How to obtain labeled proteins and what to do with them. Curr. Opin. Biotechnol..

[bib12] Huang B., Bates M., Zhuang X.W. (2009). Super-resolution fluorescence microscopy. Annu. Rev. Biochem..

[bib13] Huang F., Hartwich T.M.P., Rivera-Molina F.E., Lin Y., Duim W.C., Long J.J., Uchil P.D., Myers J.R., Baird M.A., Mothes W. (2013). Video-rate nanoscopy using sCMOS camera-specific single-molecule localization algorithms. Nat. Methods.

[bib41] Ivan R.C., Brenda B., Aihua Z., Luo S., Christopher R.P., Gra zvydas L.I., Luc R., Kai J., Ming-Qun X. (2013). Substrates for improved live-cell fluorescence labeling of SNAP-tag. Curr. Pharm. Des..

[bib42] Kapur J.N., Sahoo P.K., Wong A.K.C. (1985). A new method for gray-level picture thresholding using the entropy of the histogram. Comput. Vis. Graph Image Process.

[bib14] Keppler A., Gendreizig S., Gronemeyer T., Pick H., Vogel H., Johnsson K. (2003). A general method for the covalent labeling of fusion proteins with small molecules in vivo. Nat. Biotechnol..

[bib15] Koide Y., Urano Y., Hanaoka K., Piao W., Kusakabe M., Saito N., Terai T., Okabe T., Nagano T. (2012). Development of NIR fluorescent dyes based on Si-rhodamine for in vivo imaging. J. Am. Chem. Soc..

[bib16] Koide Y., Urano Y., Hanaoka K., Terai T., Nagano T. (2011). Evolution of group 14 rhodamines as platforms for near-infrared fluorescence probes utilizing photoinduced electron transfer. ACS Chem. Biol..

[bib43] Krieger E., Vriend G. (2015). New ways to boost molecular dynamics simulations. J. Comput. Chem..

[bib17] Kweon H.S., Beznoussenko G.V., Micaroni M., Polishchuk R.S., Trucco A., Martella O., Di Giandomenico D., Marra P., Fusella A., Di Pentima A. (2004). Golgi enzymes are enriched in perforated zones of Golgi cisternae but are depleted in COPI vesicles. Mol. Biol. Cell.

[bib18] Lang K., Davis L., Torres-Kolbus J., Chou C.J., Deiters A., Chin J.W. (2012). Genetically encoded norbornene directs site-specific cellular protein labelling via a rapid bioorthogonal reaction. Nat. Chem..

[bib19] Lang K., Davis L., Wallace S., Mahesh M., Cox D.J., Blackman M.L., Fox J.M., Chin J.W. (2012). Genetic encoding of bicyclononynes and trans-cyclooctenes for site-specific protein labeling in vitro and in live mammalian cells via rapid fluorogenic diels-alder reactions. J. Am. Chem. Soc..

[bib44] Lavieu G., Zheng H., Rothman J.E. (2013). Stapled Golgi cisternae remain in place as cargo passes through the stack. Elife.

[bib20] Liu Y., Fares M., Dunham N.P., Gao Z., Miao K., Jiang X., Bollinger S.S., Boal A.K., Zhang X. (2017). AgHalo: a facile fluorogenic sensor to detect drug-induced proteome stress. Angew. Chem. Int. Ed..

[bib21] Los G.V., Encell L.P., McDougall M.G., Hartzell D.D., Karassina N., Zimprich C., Wood M.G., Learish R., Ohane R.F., Urh M. (2008). HaloTag: a novel protein labeling technology for cell imaging and protein analysis. ACS Chem. Biol..

[bib22] Lukinavičius G., Blaukopf C., Pershagen E., Schena A., Reymond L., Derivery E., Gonzalez-Gaitan M., D’Este E., Hell S.W., Wolfram Gerlich D. (2015). SiR–Hoechst is a far-red DNA stain for live-cell nanoscopy. Nat. Commun..

[bib23] Lukinavičius G., Reymond L., D'Este E., Masharina A., Gutfert F., Ta H., Guether A., Fournier M., Rizzo S., Waldmann H. (2014). Fluorogenic probes for live-cell imaging of the cytoskeleton. Nat. Methods.

[bib24] Lukinavičius G., Reymond L., Umezawa K., Sallin O., D’Este E., Göttfert F., Ta H., Hell S.W., Urano Y., Johnsson K. (2016). Fluorogenic probes for multicolor imaging in living cells. J. Am. Chem. Soc..

[bib25] Lukinavicius G., Umezawa K., Olivier N., Honigmann A., Yang G.Y., Plass T., Mueller V., Reymond L., Correa I.R., Luo Z.G. (2013). A near-infrared fluorophore for live-cell super-resolution microscopy of cellular proteins. Nat. Chem..

[bib26] Murrey H.E., Judkins J.C., am Ende C.W., Ballard T.E., Fang Y., Riccardi K., Di L., Guilmette E.R., Schwartz J.W., Fox J.M. (2015). Systematic evaluation of bioorthogonal reactions in live cells with clickable halotag ligands: implications for intracellular imaging. J. Am. Chem. Soc..

[bib27] Nemoto Y., De Camilli P. (1999). Recruitment of an alternatively spliced form of synaptojanin 2 to mitochondria by the interaction with the PDZ domain of a mitochondrial outer membrane protein. EMBO J..

[bib45] Pettersen E.F., Goddard T.D., Huang C.C., Couch G.S., Greenblatt D.M., Meng E.C., Ferrin T.E. (2004). UCSF Chimera—a visualization system for exploratory research and analysis. J. Comput. Chem..

[bib46] Port F., Muschalik N., Bullock S.L. (2015). Systematic evaluation of *Drosophila* CRISPR tools reveals safe and robust alternatives to autonomous gene drives in basic research. G3 (Bethesda).

[bib28] Presman D.M., Ball D.A., Paakinaho V., Grimm J.B., Lavis L.D., Karpova T.S., Hager G.L. (2017). Quantifying transcription factor binding dynamics at the single-molecule level in live cells. Methods.

[bib29] Stagge F., Mitronova G.Y., Belov V.N., Wurm C.A., Jakobs S. (2013). Snap-, CLIP- and Halo-tag labelling of budding yeast cells. PLoS One.

[bib30] Takakura H., Zhang Y., Erdmann R.S., Thompson A.D., Lin Y., McNellis B., Rivera-Molina F., Uno S.-n., Kamiya M., Urano Y. (2017). Long time-lapse nanoscopy with spontaneously blinking membrane probes. Nat. Biotechnol..

[bib31] Toomre D., Bewersdorf J. (2010). A new wave of cellular imaging. Annu. Rev. Cell Dev. Biol..

[bib32] Uno S.N., Kamiya M., Yoshihara T., Sugawara K., Okabe K., Tarhan M.C., Fujita H., Funatsu T., Okada Y., Tobita S. (2014). A spontaneously blinking fluorophore based on intramolecular spirocyclization for live-cell super-resolution imaging. Nat. Chem..

[bib33] Uno S.N., Tiwari D.K., Kamiya M., Arai Y., Nagai T., Urano Y. (2015). A guide to use photocontrollable fluorescent proteins and synthetic smart fluorophores for nanoscopy. Microscopy (Oxf.).

[bib34] van de Linde S., Heilemann M., Sauer M. (2012). Live-cell super-resolution imaging with synthetic fluorophores. Annu. Rev. Phys. Chem..

[bib35] Velasco A., Hendricks L., Moremen K.W., Tulsiani D.R.P., Touster O., Farquhar M.G. (1993). Cell-type dependent variations in the subcellular-distribution of alpha-mannosidase-I and alpha-mannosidase-II. J. Cell Biol..

[bib36] Waldchen S., Lehmann J., Klein T., van de Linde S., Sauer M. (2015). Light-induced cell damage in live-cell super-resolution microscopy. Sci. Rep..

[bib37] Wodarz A., Ramrath A., Grimm A., Knust E. (2000). Drosophila atypical protein kinase C associates with bazooka and controls polarity of epithelia and neuroblasts. J. Cell Biol..

[bib38] Xue L., Karpenko I.A., Hiblot J., Johnsson K. (2015). Imaging and manipulating proteins in live cells through covalent labeling. Nat. Chem. Biol..

