# Supplementary material for: Labeling Strategies Matter for Super-Resolution Microscopy: A Comparison between HaloTags and SNAP-tags
Source: Cell Chem Biol. 2019 Apr 18;26(4):584–592.e6. doi: 10.1016/j.chembiol.2019.01.003 (PMC6474801; doi:10.1016/j.chembiol.2019.01.003)
Supplement: Document S1. Figures S1–S9 and Table S1 [file mmc1.pdf]

Cell Chemical Biology, Volume *26*

Supplemental Information

Labeling Strategies Matter for Super-Resolution Microscopy: A Comparison between HaloTags and SNAP-tags

Roman S. Erdmann, Stephanie Wood Baguley, Jennifer H. Richens, Rebecca F. Wissner, Zhiqun Xi, Edward S. Allgeyer, Sheng Zhong, Alexander D. Thompson, Nicholas Lowe, Richard Butler, Joerg Bewersdorf, James E. Rothman, Daniel St Johnston, Alanna Schepartz, and Derek Toomre

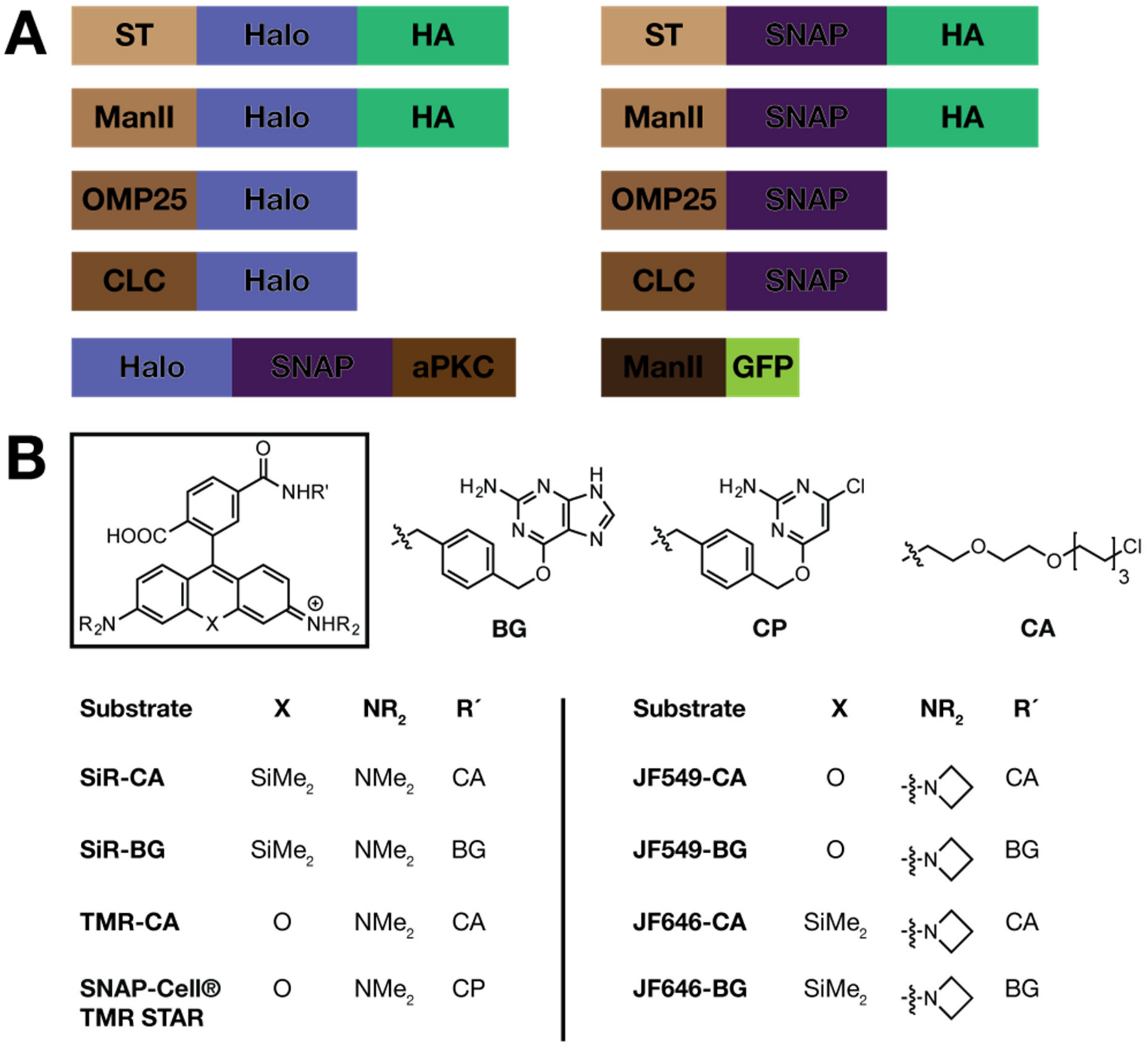

Figure S1 (related to Figures 1-4). Overview of self-labeling fusion proteins (A) and corresponding substrates (B) used in this study. Ligands reacting with HaloTag (R'=CA) are referred to as dye-HaloTag ligand in the text and ligands reacting with SNAP-tag (R'=BG or CP) are referred to as dye-SNAP-tag ligand in the test.

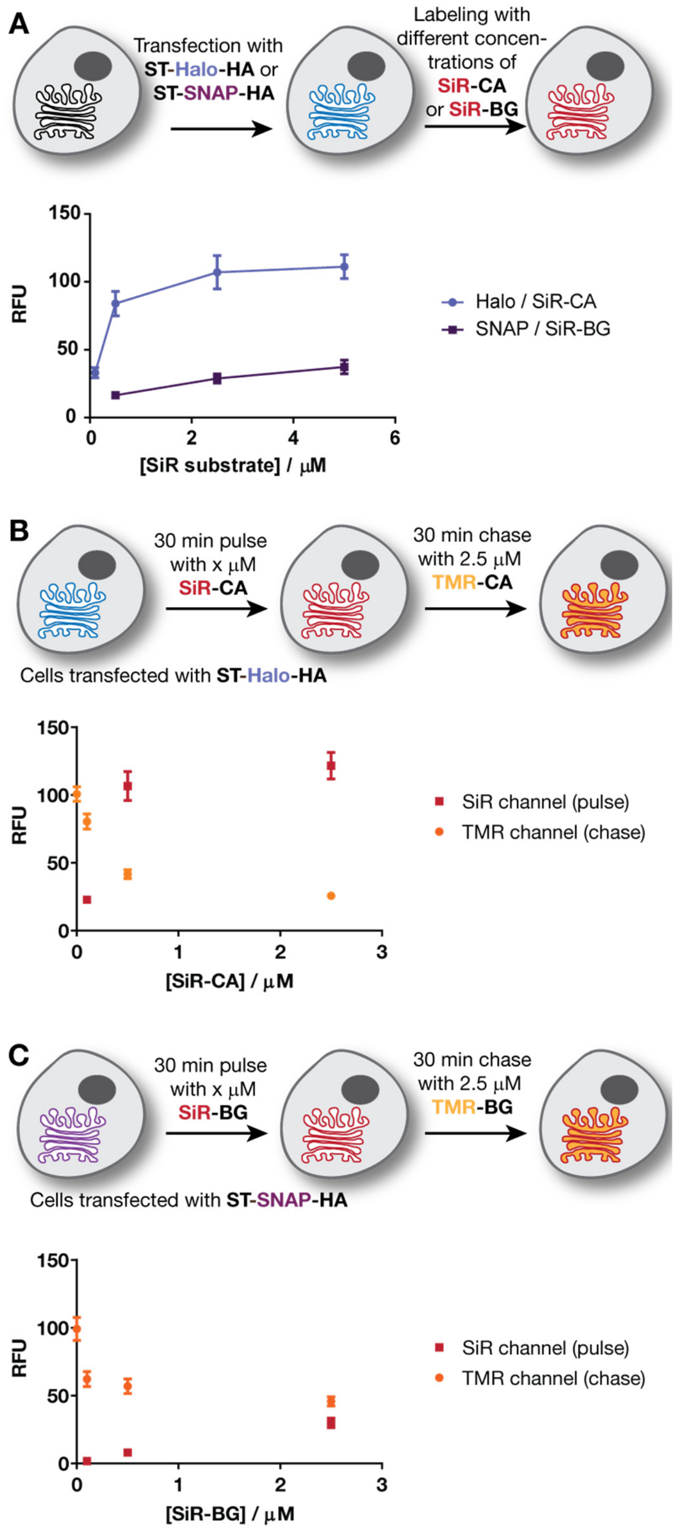

Figure S2 (related to Figure 2). A) Comparison between SNAP and Halo labeling intensity with different concentrations of SiR-substrates. B) Labeling intensities of Halo-tag after a pulse labeling with SiR substrate at different concentrations followed by a chase labeling with 2.5 µM TMR-substrate. C) Labeling intensities of SNAP-tag after a pulse labeling with SiR substrate at different concentrations followed by a chase labeling with 2.5 µM TMR-substrate. Error bars show SEM.

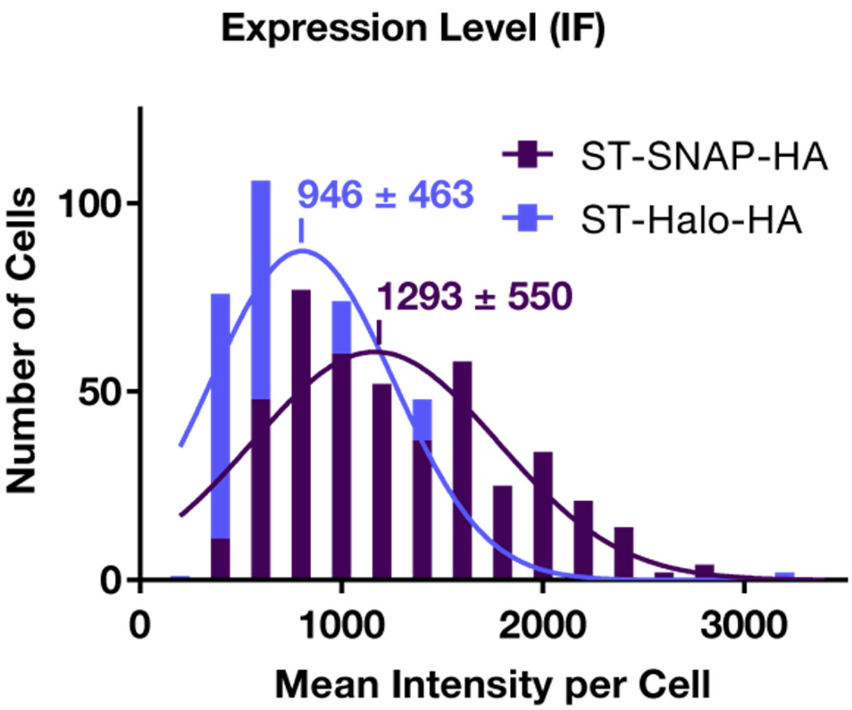

Figure S3 (related to Figure 2). Intensity distribution in HeLa cells that are transiently expressing ST-SNAP-HA (n=443) or ST-Halo-HA (n=481) and have been immunostained with and anti-HA antibody followed by an Alexa Fluor 546 labeled goat anti-mouse antibody. The fluorescence intensity serves as a measure of expression level.

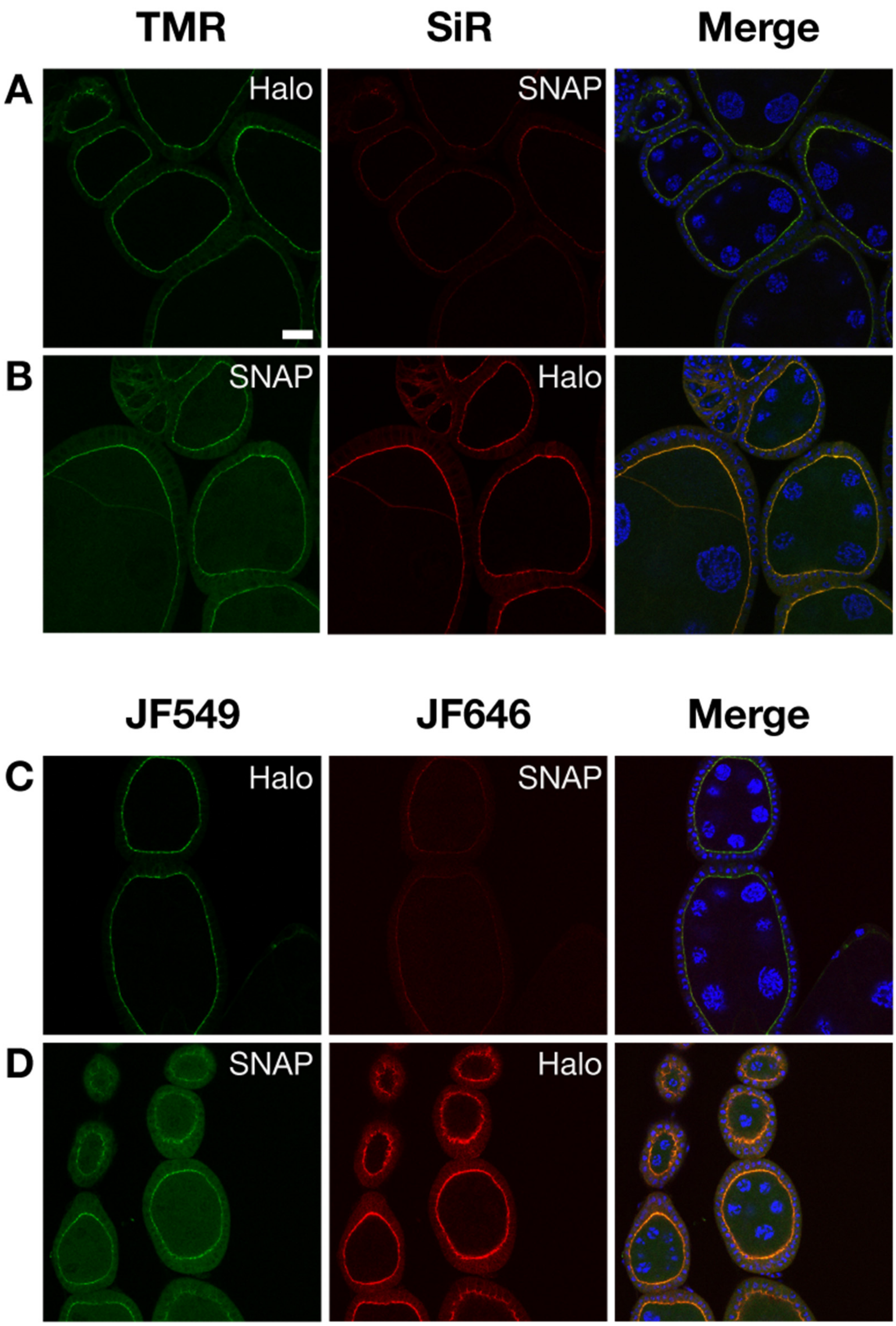

Figure S4 (related to Figure 3). A-D) Halo-SNAP-aPKC egg chambers, stained with DAPI (in the merge) and A) TMR-CA and SiR-BG, B) SNAP-Cell® TMR-Star and SiR-CA, C) JF549-CA and JF646-BG, D) JF549-BG and JF646-CA. A & B) Images were taken, and are displayed, with identical settings. C & D) Images were taken, and are displayed, with identical settings. Scale bar is 20 µm.

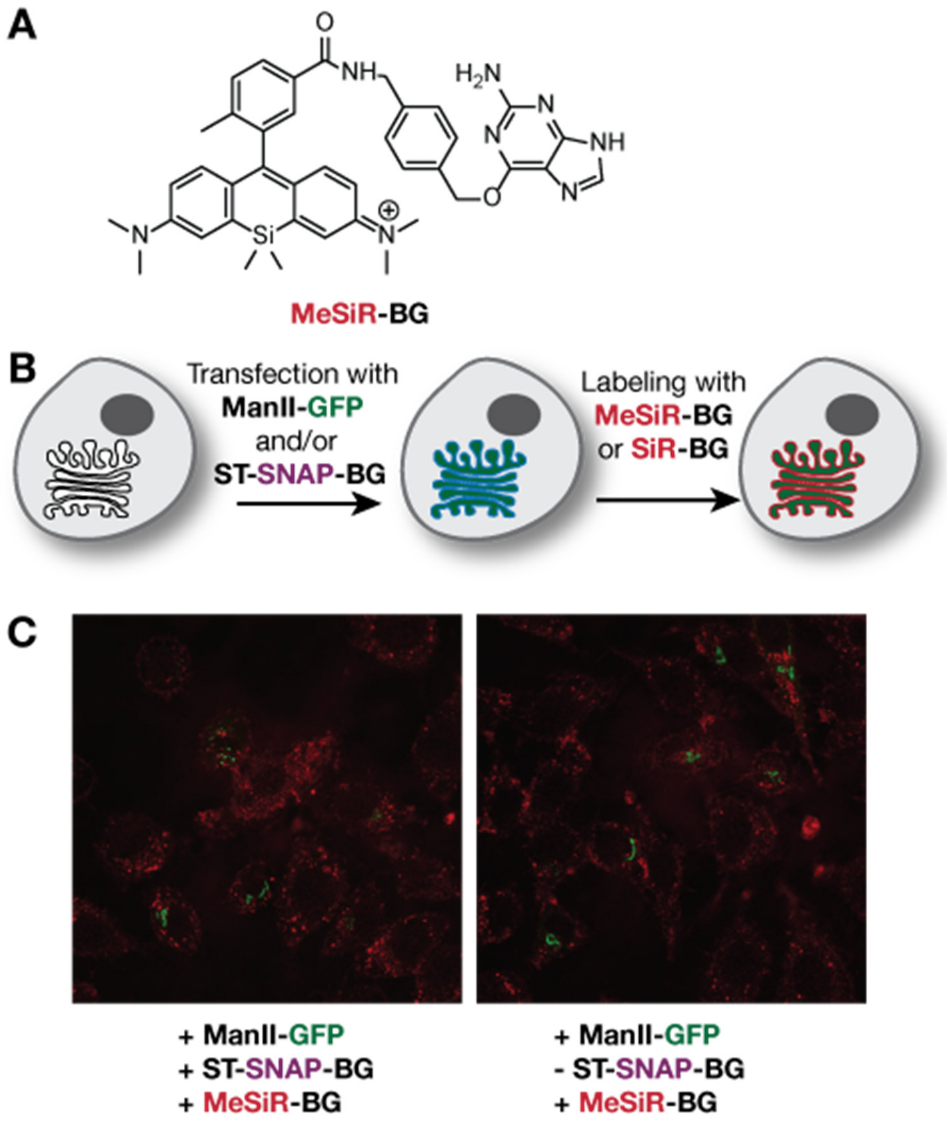

Figure S5 (related to Figure 3). A) Chemical Structure of MeSiR-BG. B) Labeling procedure with and without transfection with ST-SNAP-HA. C) Images of cells labeled with MeSiR-BG in presence and absence of transfection with ST-SNAP-HA.

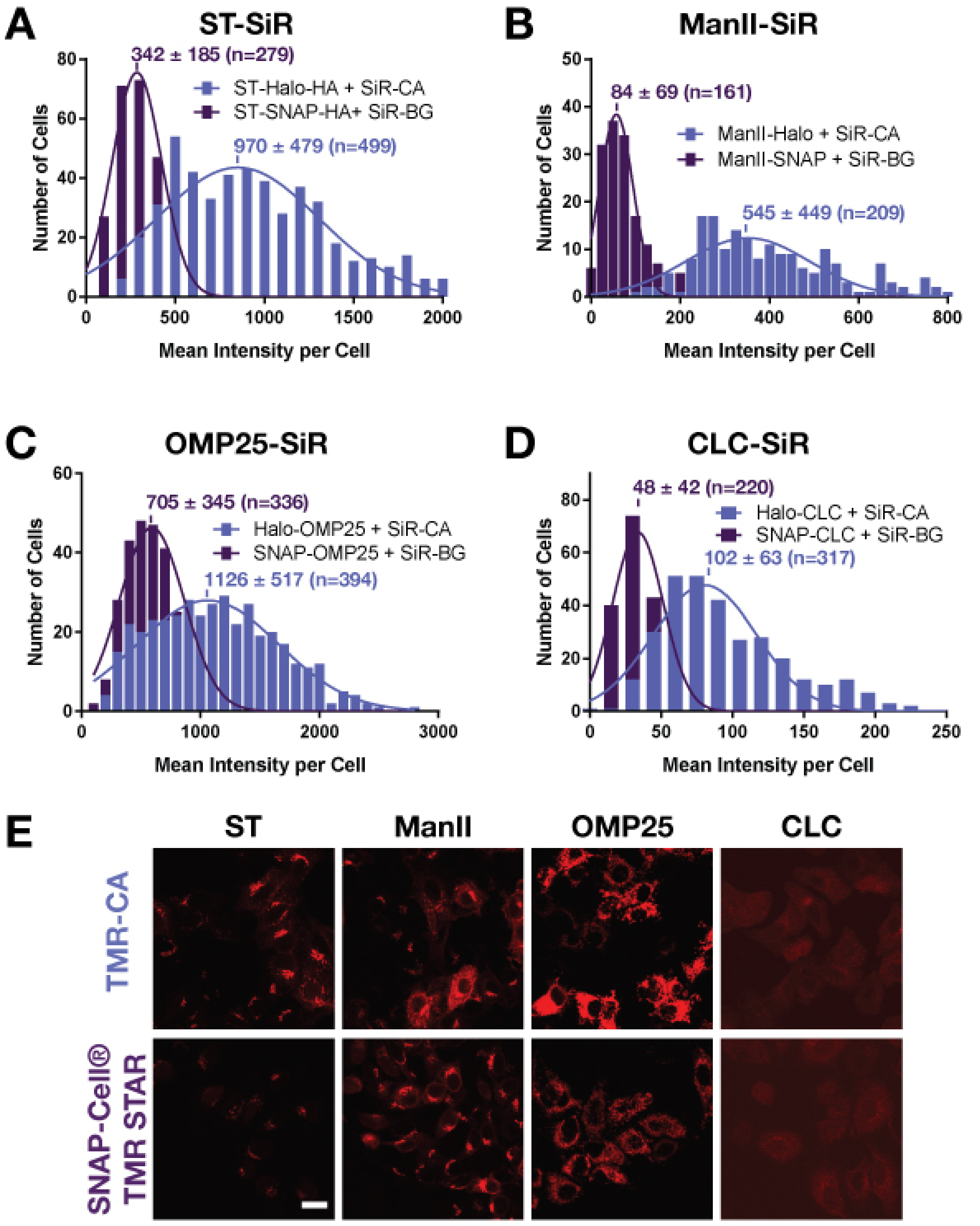

Figure S6 (related to Figure 3). A-D) Intensity distribution in HeLa cells that are transiently expressing various self-labeling fusion proteins that have been labeled with either SiR-CA or SiR-BG as shown in Figure 3A. E) Confocal images of HeLa cells in which various targets were labeled with SiR-CA or SiR-BG using a SNAP-tag or Halo-tag labeling strategy, respectively. Images of SNAP and Halo-tagging of the same target were acquired and are displayed with identical settings. Scale bar is 20 μm.

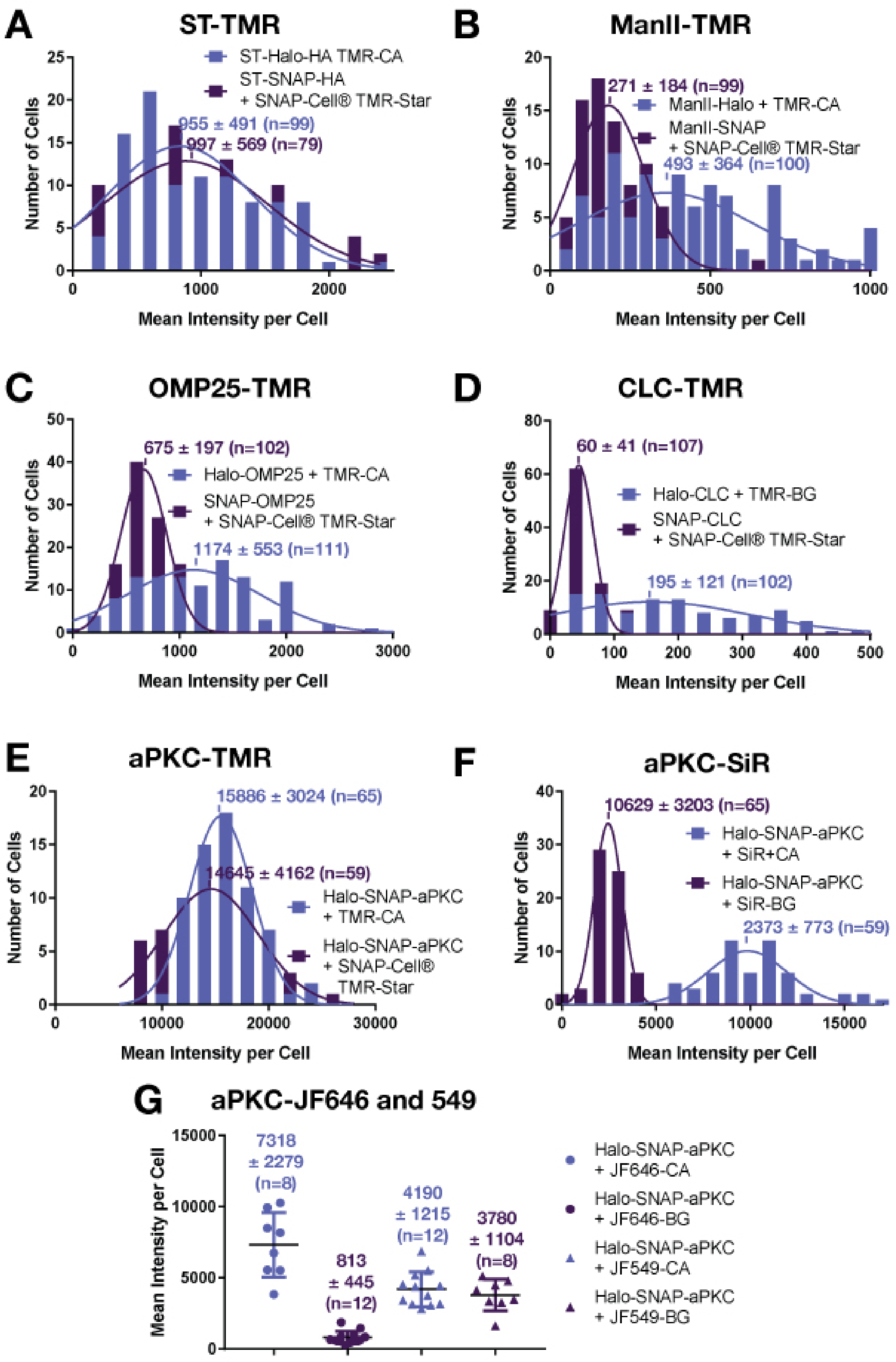

Figure S7 (related to Figure 3). A-D) Intensity distribution in HeLa cells that are transiently expressing various self-labeling fusion proteins that have been labeled with either TMR-CA or SNAP-Cell® TMR-Star is shown in Figure S6E7. E-G) Intensity distribution in Drosophila egg chambers that are expressing Halo-SNAP-aPKC and have been labeled with either various Halo and SNAP substrates as shown in Figure 3D and S4.

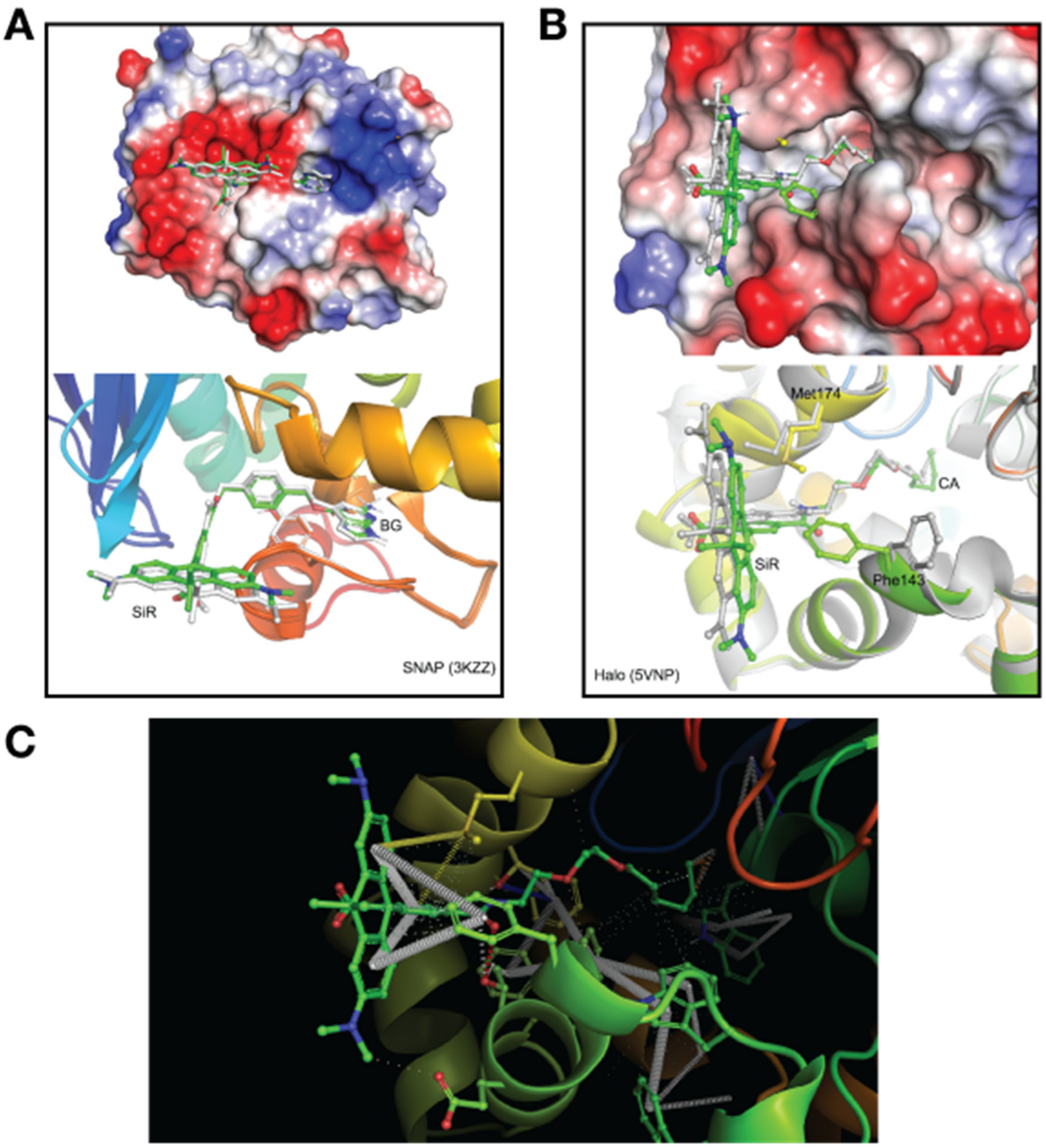

Figure S8 (related to Figure 1-4). Interactions of SiR with Halo and SNAP. A) Electrostatic surface of SNAP (pdb 3KZZ) conjucated with SiR-BG (top) and the tertiary structure of the SNAP binding site (bottom). Interaction analysis did not find any obvious interaction between SNAP and SiR part, and the energy minimization does not change the orientation of SiR or any residue.

(B) Electrostatic surface of Halo (pdb 5VNP) interacting with a SiR-CA (top) and tertiary structure showing the change in orientation of Phe143 and Met174 due to the interaction with SiR (bottom). The gray structures and residues are before energy minimization and the colored structure and residues (Phe143 and Met174) are after energy minimization. The aromatic interaction on the conjugated structure of SiR could directly affect the molecular orbital energy (HOMO & LUMO) that may change the optical properties of SiR indeed. C) Interactions between SiR-CA and residues Phe143 and Met 174.

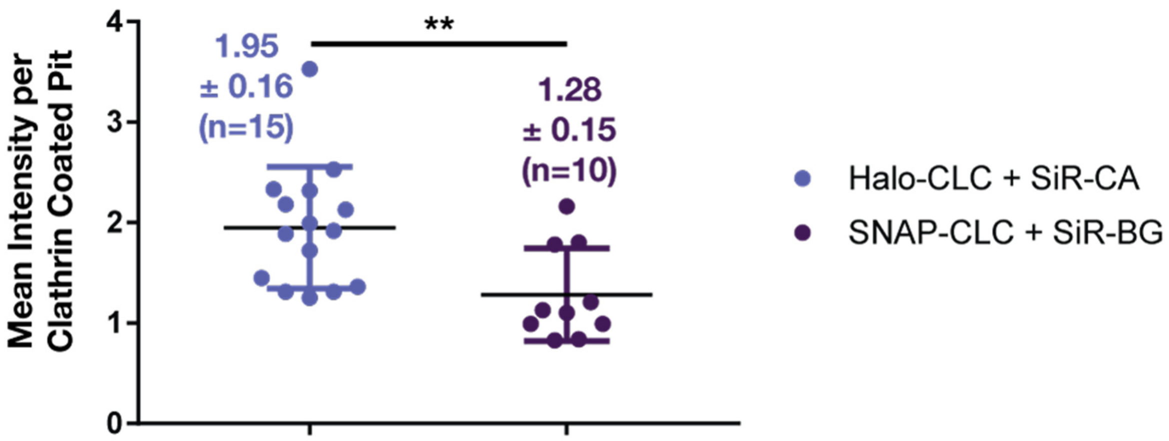

Figure S9 (related to Figure 4). Intensity distribution in STED images of HeLa cells that are transiently expressing various Halo-CLC or SNAP-CLC and have been labeled with either SiR-CA or SiR-BG, respectively (as shown in Figure 4D).

	λ_{max} / $\lambda_{em,max}$ [nm]	ε_{max} [M^{-1} cm^{-1}]	**Quantum yield**	**Relative brightness**
SiR carboxyl	645* / 661*	100,000*	0.39*	3.00
SiR-BG	650 / n/d	10,100	n/d	n/d
SiR-CA	647 / n/d	8,300	n/d	n/d
SiR-Halo	648* / 668*	130,200	0.39*	3.92
SiR-SNAP	650* / 668*	43,200	0.30*	1.00

Table S1 (related to Figure 1-4). Photophysical properties of different SiR conjugates. Relative brightness was calculated as product of quantum yield and extinction coefficient and normalized to SiR-SNAP. * values taken from reference Lukinavicius et al., 2013.
